# Effect of Dietary Rumen-Protected Methionine and Lysine on Performance and Plasma Amino Acid of Dairy Cows: A Meta-Analysis

**DOI:** 10.3390/ani16213442

**Published:** 2026-10-31

**Authors:** Fujiang Hu, Jiahao Xiao, Yuancheng Guo, Yang Mi, Rong Huang, Linsen Zan, Wucai Yang, Chunping Zhao

**Affiliations:** College of Animal Science and Technology, Northwest A&F University, Yangling, Xianyang 712100, China; 2025055525@nwafu.edu.cn (F.H.); 2025055502@nwafu.edu.cn (J.X.);

**Keywords:** meta-analysis, dairy cow, methionine, lysine, lactation performance

## Abstract

Low-protein diets supplemented with rumen-protected methionine and lysine (RPML) can optimize amino acid supply while maintaining milk performance and reducing environmental impacts. But responses of cows to RPML are inconsistent and modulating factors remain poorly understood. This meta-analysis tested whether RPML supplementation improves lactational performance and alters plasma amino acid profiles of dairy cows. Forty-two eligible studies published up to 1 June 2026 were retrieved from PubMed, Web of Science and ScienceDirect. Meta-analysis, subgroup analysis, correlation analysis and dose–response regression were adopted. Results showed that RPML supplementation significantly increased milk yield by 0.500 kg/d, energy-corrected milk by 1.378 kg/d, protein yield by 0.038 kg/d, protein concentration by 0.052%, fat yield by 0.030 kg/d, fat concentration by 0.043%, 3.5% fat-corrected milk by 0.778 kg/d, milk total nitrogen by 0.010%, and casein nitrogen by 0.007%. Plasma arginine, asparagine, isoleucine, leucine, lysine and methionine increased, while glycine declined. Subgroup analysis showed that cow breed, parity, lactation stage, basal diet and dietary crude protein were key moderators. Digestible methionine dosage correlated with milk fat concentration and plasma leucine, and digestible lysine dosage correlated with milk protein yield. Significant regression models confirmed the combined effects of methionine and lysine on multiple milk traits. These results indicate that supplementation with RPML represents an effective nutritional strategy for enhancing milk yield and milk quality in dairy cows. Such findings may assist producers and nutritionists in refining amino acid feeding programs to optimize dairy cow productive performance.

## Simple Summary

High-protein diets help cow milk output but waste nitrogen and pollute the environment. Using lower-protein feeds plus protected methionine and lysine can fix these problems, but response to RPML are inconsistent across studies. To test whether RPML supplementation improves lactational performance and alters plasma amino acid profiles of dairy cows, this meta-analysis synthesized data from 42 studies related to dairy cow fed RPML. Supplementing RPML increased daily milk yield, energy-corrected milk, milk protein yield, fat yield, protein concentration, fat concentration, total milk nitrogen and casein nitrogen. Amino acids in cow blood (arginine, asparagine, isoleucine, leucine, lysine and methionine) increased, whereas glycine decreased. Further analysis showed that cow breed, parity, lactation stage, basal diet and dietary crude protein level changed how cows responded to the RPML supplements. Digestible methionine dosage correlated with milk fat concentration and plasma leucine, and digestible lysine dosage correlated with milk protein yield. Regression models indicated that combined supplementation of methionine and lysine worked together to improve milk performance. In summary, our results support that adding RPML is an effective nutritional strategy to boost milk production and milk quality in dairy cows, and these findings can help farmers and nutritionists better design amino acid feeding practice.

## 1. Introduction

To increase milk yield and quality, dairy farmers commonly adopt high-concentrate, especially high-protein diets. However, these feeding strategies reduce nitrogen (N) utilization efficiency and correspondingly increase N excretion in feces and urine, resulting in environmental pollution [1]. Consequently, low-protein diet strategies have emerged through reducing soybean meal inclusion, along with supplementing limiting AAs (amino acids) to achieve AA balance in dairy cow diets, thereby maintaining or even enhancing milk performance while lowering production costs and N emissions [2]. Met and Lys serve as the first and second limiting AAs in dairy cow production, playing pivotal roles in physiological metabolism and regulation of production performance [3]. Given that both AAs are susceptible to degradation by rumen microorganisms, rumen-protected Met and Lys (RPML) are employed in practical ruminant production to enhance their intestinal absorption and utilization [4]. Supplementation of RPML enhances protein synthesis and antioxidant capacity in dairy cows while alleviating oxidative stress, ultimately improving metabolic health and production performance [5].

Some studies have explored the effects of supplementing dairy cattle with Met [6] and His [7], RPL [8], or RPM [9] on their performance. But the above-mentioned studies did not treat RPM and RPL as two parallel dose variables in a joint model. A study reviewed the percentage changes in the performance of lactating dairy cows supplemented with Met and Lys [10]. Importantly, numerous studies have examined the effects of dietary RPML supplementation in dairy cows. Yet, findings remain inconsistent: some research indicates that RPML significantly improves production performance, whereas others report negligible effects. Meta-analysis offers a robust methodological approach to synthesize findings across multiple studies on the same research theme and generates more reliable conclusions [11]. Previous meta-analyses on this topic have some notable limitations. A meta-analysis pooled data from post-ruminal infusion and rumen-protected administration without accounting for divergent absorption kinetics between these delivery routes [12]. Additionally, this study did not report results from trials with both Met and Lys supplementation, thereby neglecting their potential combining or antagonistic interactions. Furthermore, publication bias was not assessed in this study, potentially leading to over-representation of positive findings. Another meta-analysis was restricted to diets with crude protein (CP) ≤ 150 g/kg of DM and failed to report critical methodological details [13]. Additionally, Irawan et al. compared the effect of RPML supplementation to Lys on milk production, but they did not adequately explore potential covariates [3].

Based on these considerations, the primary objectives of this study were to systematically evaluate the effects of RPML supplementation on dairy cow performance and plasma AA concentrations and to analyze key influencing factors for effect sizes comprehensively. The secondary objective was to quantify dose–response relationships between these two essential AAs and corresponding traits. Therefore, this study will provide a scientific basis for the efficient utilization of AA additives in dairy cow production.

## 2. Materials and Methods

### 2.1. Literature Search

This study was conducted in accordance with the Preferred Reporting Items for Systematic Reviews and Meta-Analyses (PRISMA) guidelines [14]. Three researchers independently searched PubMed, ScienceDirect, and Web of Science for eligible studies published before June 1, 2026. The search strategy employed the following in all three datasets: “Lys” OR “Lysine” OR “Met” OR “Methionine” AND “cattle” OR “cow”. The researchers also identified additional relevant publications through manual screening of reference lists. The study selection process is illustrated in Figure S1.

The inclusion criteria were as follows: (1) inclusion of a control group without RPML supplementation; (2) random allocation of cows to treatments; (3) reporting of variation and relevant parameters (i.e., sample size, mean, and standard deviation); (4) for studies involving both RPML and other supplements in the same publication, we included the comparison between the RPML supplemented group and the non-RPML one; and (5) the RPML was added as a form of feeding and not infused into the abomasum, duodenum, or jugular vein. The exclusion criteria included the following: (1) studies without RPML data or corresponding trait data; (2) studies with the data from non-dairy cattle; (3) studies with RPML combined with other additives; (4) corresponding traits and data missing; (5) grazing cows; (6) review and meta-analysis; and (7) RPML infused into the abomasum, duodenum or jugular vein.

### 2.2. Data Extraction

The following information was extracted: first author, year, country, cattle breed, lactation stage, sample size, additive composition, experiment duration, diet composition, supplementation methods, and mean, standard deviation or standard error of all traits corresponding to RPML and control groups. The standard error for traits was converted to standard deviation using the formula ($SD=SEM\times \sqrt{n})$. The main traits were sorted into three categories: body performance (BW (body weight), DMI (dry matter intake), BCS (body condition score)); lactation performance (milk yield (kg/d), ECM (energy-corrected milk) (kg/d), protein yield (kg/d) and concentration (%), fat yield (kg/d) and concentration (%), 3.5% FCM (fat-corrected milk) (kg/d), lactose yield (kg/d) and concentration (%), MUN (milk urea nitrogen) (mg/dL), total N (%), casein N (%), Whey N (%)); and plasma AA concentrations (μmol/L).

The possible sources of variability, such as cow breed, parity, lactation stage, silage type, and CP level of diets, dosages for digestible Met (*dMet*) and digestible Lys (*dLys*), were also extracted or calculated.

### 2.3. Methods for Quality and Risk of Bias Assessment

Two researchers independently assessed study quality following the criteria in the Cochrane Collaboration’s tool and the statement of Consolidated Standards of Reporting Trials (Version 6.5) [15]. Disagreements in the assessment were resolved through discussions with a third researcher.

### 2.4. Statistical Analyses

For the meta-analysis, the raw mean difference (RMD) between the RPML treatment and control groups was estimated with 95% confidence intervals (CIs) using a random-effects model [6]. The pooled effects were obtained by the inverse-variance method, with the between-study variance estimated using restricted maximum likelihood. The Wald-type test was applied when constructing 95% CIs. Statistical heterogeneity was assessed using the I^2^ statistic. All analyses were performed in Stata 18.0 using the metan package (version 1.85) [16]. Subgroup analyses were performed for affected traits (*P_RMD_* < 0.1 and I^2^ > 50%) to explore the sources of heterogeneity, including breed (Holstein or other breed), parity (multiparous or mixed parity), silage type of basic diets (corn silage, grass silage, or mixed silage), lactation stage (pre, early (≤120 DIM (days in milk)), or mid (120 < DIM < 240) [6], CP level of diets (low (CP < 15%) or high (CP ≥ 15%)). Mantel test and Pearson correlation analysis were performed to assess the relationship of RPML and the significantly affected traits using the ggplot2 and LinkET package in R software (Version 4.5.1) [17,18]. In the Mantel analysis, Bray–Curtis distance matrices were calculated separately for the two amino acid groups (*dMet* and *dLys*) while Euclidean distance matrices were computed for each of the significantly affected traits in subgroup analysis. Finally, we performed regression analysis to explore the dose–response relationship among *dMet*, *dLys*, and significantly affected traits in subgroup analysis using the “rsm” package in R software and assessed model adequacy via adjusted R^2^ and model *p*-value [19].

### 2.5. Methods for Publication Bias and Sensitivity Analysis

Publication bias was examined using Egger’s linear regression test [20]. Sensitivity analysis was performed by iteratively omitting one study at a time to evaluate the influence of individual studies on the overall effect size. Statistical significance was set at *p* < 0.05 [21].

## 3. Results

### 3.1. Study Selection and Characteristics

A total of 3374 publications were initially retrieved from the three databases. After removing duplicates and excluding studies unrelated to cattle, involving non-RPML or multiple supplements, grazing, etc., 31 papers remained. We also identified additional 11 papers from relevant meta-analyses and reference lists. Ultimately, we selected 42 papers that met the criteria and extracted corresponding data to construct the database. The process of retrieving, identifying, and excluding studies is shown in Figure S1.

All the trials included Holstein (*n* = 35) and other breeds (*n* = 7), with multiparous (*n* = 28), mixed (primiparous and multiparous) parity (*n* = 13), and primiparous cows (*n* = 1); the primiparous group was not included in subgroup analysis. The cows were sorted into pre- (*n* = 6), early (*n* = 30), and mid-lactation (*n* = 7) due to one publication including both early and mid-lactation stage cows. Main dietary forages were corn silage (*n* = 27), grass silage (*n* = 5), and mixed silage (*n* = 11) due to one publication including both corn silage and mixed silage. The diets in the studies included low-CP (*n* = 15) and high-CP diets (*n* = 30) due to three publications including both low- and high-CP diets. The descriptive statistics of each outcome are shown in Table 1.

### 3.2. Quality and Risk of Bias Assessment

**Figure 1:**
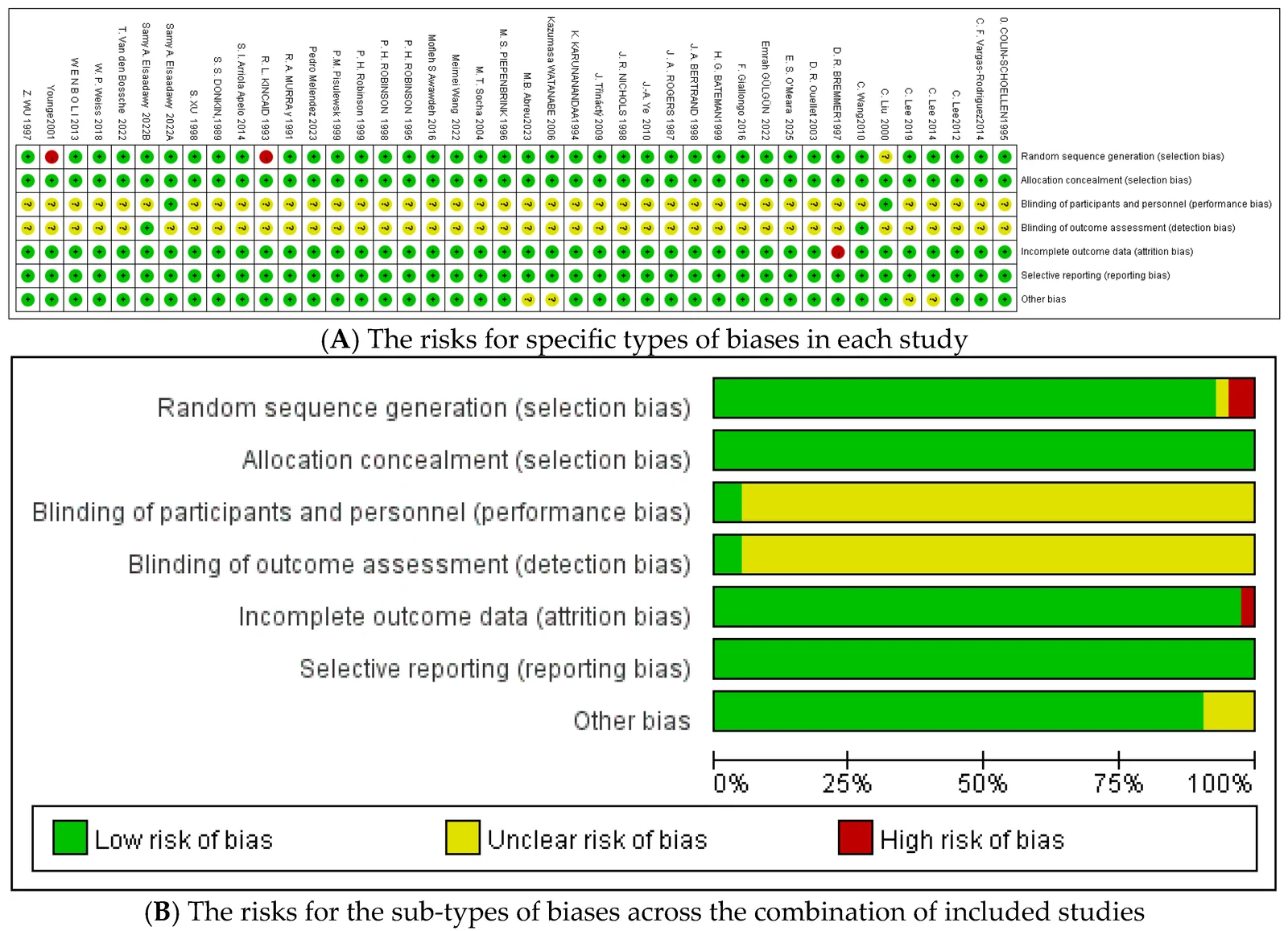
Quality and risk of bias assessment of all included studies. (**A**) The risks for specific types of biases in each study, with - indicating high risk, + indicating low risk, and ? indicating unclear risk. (**B**) The risks for the sub-types of biases across the combination of included studies, with colors indicating bias risk levels: green for low risk, yellow for unclear risk, and red for high risk.

The risks of bias for each study and overall are shown visually in Figure 1. In the first domain (random sequence generation), two trials were classified as high risk of bias and one as unclear risk. In the second domain (allocation concealment) and sixth domain (selective reporting), all trials were classified as low risk of bias. In the third (blinding of participants and personnel) and fourth domain (blinding of outcome assessment), two trials were classified as low risk of bias and others were unclear risk, respectively. In the fifth domain (incomplete outcome data), one trial was classified as high risk, and the other as low risk. In the seventh domain (other bias), four trials were classified as unclear risk while the other was classified as low risk. Overall, the low risk of bias and acceptable quality from the studies examined largely support our further meta-analysis.

### 3.3. Effects of RPML Supplementation on Body Performance

The summary of the meta-analysis on the effects of RPML on the body performance of dairy cows is shown in Table 2. RPML did not significantly affect BW, DMI and BCS (*p* > 0.05).

### 3.4. Subgroup Analysis of Effects of RPML Supplementation on DMI

Further subgroup analysis was performed on DMI because there was a trend for RPML to affect DMI (*p* = 0.085). The results showed that RPML supplementation significantly increased the DMI of Holstein by 0.378 kg/d (*p* = 0.037) whilst decreasing the DMI of other breeds by 0.379 kg/d (*p* = 0.014). Additionally, the DMI was significantly increased when RPML was supplied to cows at the pre-lactation stage (RMD = 0.576 kg/d, *p* = 0.017). Still, DMI was not affected when RPML was supplied at the early or mid-lactation stage (*p* > 0.05) (Table S1).

### 3.5. Effects of RPML Supplementation on Lactation Performance

RPML significantly increased milk yield (RMD = 0.500 kg/d, *p* = 0.001), ECM (RMD = 1.378 kg/d, *p* = 0.003), protein yield (RMD = 0.038 kg/d, *p* < 0.001), protein concentration (RMD = 0.052%, *p* < 0.001), fat yield (RMD = 0.030 kg/d, *p* = 0.002), fat concentration (RMD = 0.043%, *p* = 0.009), 3.5% FCM (RMD = 0.778 kg/d, *p* = 0.003), total N (RMD = 0.010%, *p* = 0.009), and casein N (RMD = 0.007%, *p* = 0.045). However, RPML did not significantly affect milk lactose yield and concentration, MUN, and Whey N (*p* > 0.05) (Table 3).

### 3.6. Subgroup Analysis of Effects of RPML Supplementation on Lactation Performance

#### 3.6.1. Milk Yield

Holstein cows (RMD = 0.658 kg/d, *p* < 0.001), multiparous cows (RMD = 0.552 kg/d, *p* = 0.001), cows in pre-lactation (RMD = 1.268 kg/d, *p* = 0.006), in early lactation (RMD = 0.670 kg/d, *p* < 0.001), fed corn silage (RMD = 0.486 kg/d, *p* = 0.013), and fed a high-CP diet (RMD = 0.952 kg/d, *p* < 0.001) significantly increased milk yield responding to RPML Supplementation (Table S2).

#### 3.6.2. ECM

Multiparous cows (RMD = 2.418 kg/d, *p* = 0.001), those in pre-lactation (RMD = 1.882 kg/d, *p* = 0.001), and fed grass silage (RMD = 3.667 kg/d, *p* < 0.001) had a significantly increased ECM responding to RPML Supplementation (Table S2).

#### 3.6.3. Milk Protein Yield

Holstein cows (RMD = 0.045 kg/d, *p* < 0.001), multiparous cows (RMD = 0.047 kg/d, *p* < 0.001), those in pre-lactation (RMD = 0.065 kg/d, *p* < 0.001), and in early lactation (RMD = 0.045 kg/d, *p* < 0.001) had a significant increase in protein yield to RPML supplementation. Additionally, cows fed either corn silage (RMD = 0.037 kg/d, *p* < 0.001), grass silage (RMD = 0.040 kg/d, *p* < 0.001), or mixed silage (RMD = 0.031 kg/d, *p* = 0.003), or fed either a low-CP diet (RMD = 0.029 kg/d, *p* = 0.001) or a high-CP diet (RMD = 0.047 kg/d, *p* < 0.001), had a significant increase in protein yield response to RPML Supplementation (Table S2).

#### 3.6.4. Milk Protein Concentration

Cows in early lactation (RMD = 0.065%, *p* < 0.001), in mid-lactation (RMD = 0.035%, *p* = 0.004), and fed corn silage (RMD = 0.060%, *p* < 0.001) had a significantly increased milk protein concentration responding to RPML supplementation. Additionally, either Holstein cows (RMD = 0.049%, *p* < 0.001) or other breeds (RMD = 0.076%, *p* = 0.035), either multiparous (RMD = 0.055%, *p* < 0.001) or mixed parity (RMD = 0.053%, *p* = 0.020), or those either fed a low-CP diet (RMD = 0.051%, *p* < 0.001) or a high-CP diet (RMD = 0.050%, *p* < 0.001) had a significant increase in milk protein concentration response to RPML Supplementation (Table S2).

#### 3.6.5. Milk Fat Yield

Holstein cows (RMD = 0.034 kg/d, *p* = 0.002), multiparous cows (RMD = 0.038 kg/d, *p* = 0.001), those in pre-lactation (RMD = 0.075 kg/d, *p* < 0.001), in early lactation (RMD = 0.023 kg/d, *p* = 0.049), fed grass silage (RMD = 0.045 kg/d, *p* = 0.014), and fed a high-CP diet (RMD = 0.047 kg/d, *p* = 0.001) had a significantly increased fat yield responding to RPML Supplementation (Table S2).

#### 3.6.6. Milk Fat Concentration

Holstein cows (RMD = 0.039%, *p* = 0.025), those fed mixed silage (RMD = 0.052%, *p* = 0.010), and fed a high-CP diet (RMD = 0.063%, *p* = 0.007) had a significantly increased milk fat concentration responding to RPML supplementation. Additionally, parity and lactation stage had no effect on it (*p* > 0.05) (Table S2).

#### 3.6.7. The Yield of 3.5% FCM

Multiparous cows (RMD = 0.848 kg/d, *p* = 0.003), those in pre-lactation (RMD = 2.659 kg/d, *p* < 0.001), fed grass silage (RMD = 4.083 kg/d, *p* < 0.001), and fed a high-CP diet (RMD = 1.589 kg/d, *p* < 0.001) had a significantly increased 3.5% FCM responding to RPML Supplementation (Table S2).

#### 3.6.8. Total N, Casein N, Whey N

Furthermore, cows either fed corn silage (RMD = 0.011%, *p* = 0.042) or grass silage (RMD = 0.011%, *p* = 0.005) had a significant increase in total N concentration response to RPML supplementation; cows fed grass silage had a significantly higher casein N (RMD = 0.010%, *p* = 0.013); and Holstein cows had a significantly increased milk Whey N concentration (RMD = 0.003%, *p* = 0.047) (Table S2).

### 3.7. Effects of RPML Supplementation on Plasma AAs

RPML significantly increased plasma concentrations of Arg (RMD = 4.071 μmol/L, *p* = 0.001), Asn (RMD = 1.693 μmol/L, *p* = 0.015), Ile (RMD = 5.641 μmol/L, *p* < 0.001), Leu (RMD = 5.325 μmol/L, *p* = 0.007), Lys (RMD = 10.112 μmol/L, *p* < 0.001), and Met (RMD = 8.242 μmol/L, *p* < 0.001), while significantly decreasing Gly (RMD = −14.408 μmol/L, *p* = 0.006). However, RPML did not significantly affect plasma concentrations of Ala, Asp, Gln, Glu, His, Phe, Pro, Ser, Thr, Trp, Tyr, and Val (*p* > 0.05) (Table 4).

### 3.8. Subgroup Analysis of Effects of RPML Supplementation on Plasma AAs

#### 3.8.1. Arg

RPML supplementation significantly increased plasma Arg in Holstein cows (RMD = 3.637 μmol/L, *p* = 0.002), mixed-parity cows (RMD = 5.772 μmol/L, *p* < 0.001), and those at the early-lactation stage (RMD = 5.910 μmol/L, *p* < 0.001). Arg was increased by RPML regardless of silage type and CP level in diets (*p* < 0.05 for each subgroup) (Table S3).

#### 3.8.2. Asn

RPML supplementation significantly increased plasma Asn in cows of mixed parity (RMD = 2.783 μmol/L, *p* = 0.003), in pre-lactation (RMD = 2.834 μmol/L, *p* = 0.016), and fed a low-CP diet (RMD = 2.836 μmol/L, *p* = 0.010) (Table S3).

#### 3.8.3. Gly

RPML supplementation significantly decreased plasma Gly in Holstein cows (RMD = −14.644 μmol/L, *p* = 0.017), multiparous cows (RMD = −25.419 μmol/L, *p* < 0.001), those in the mid-lactation stage (RMD = −18.428 μmol/L, *p* = 0.003), fed corn silage (RMD = −19.415 μmol/L, *p* = 0.001), and fed a low-CP diet (RMD = −17.743 μmol/L, *p* = 0.027) (Table S3).

#### 3.8.4. Ile

RPML supplementation significantly increased plasma Ile in Holstein cows (RMD = 5.833 μmol/L, *p* < 0.001), those in early lactation (RMD = 6.696 μmol/L, *p* < 0.001), in mid-lactation (RMD = 5.567 μmol/L, *p* = 0.042), and fed a low-CP diet (RMD = 8.202 μmol/L, *p* < 0.001). Ile was increased by RPML regardless of parity and basic diet (*p* < 0.05 for each subgroup) (Table S3).

#### 3.8.5. Leu

RPML significantly increased plasma Leu in Holstein cows (RMD = 5.543 μmol/L, *p* = 0.004), multiparous cows (RMD = 5.290 μmol/L, *p* = 0.040), those in early lactation (RMD = 6.193 μmol/L, *p* = 0.031), fed corn silage (RMD = 4.860 μmol/L, *p* = 0.027), and fed a low-CP diet (RMD = 5.818 μmol/L, *p* = 0.024) (Table S3).

#### 3.8.6. Lys

RPML significantly increased plasma Lys in Holstein (RMD = 10.711 μmol/L, *p* < 0.001). Lys was increased by RPML regardless of parity, lactation stage, basic diet and CP level in diets (*p* < 0.05 for each subgroup) (Table S3).

#### 3.8.7. Met

RPML significantly increased plasma Met in cows in the early (RMD = 8.757 μmol/L, *p* < 0.001) and mid-lactation stages (RMD = 11.179 μmol/L, *p* < 0.001). Met was increased by RPML regardless of breed, parity, basic diet and CP level in diets (*p* < 0.05 for each subgroup) (Table S3).

### 3.9. Correlation Analysis of RPML Supplementation in Holstein Cows

**Figure 2:**
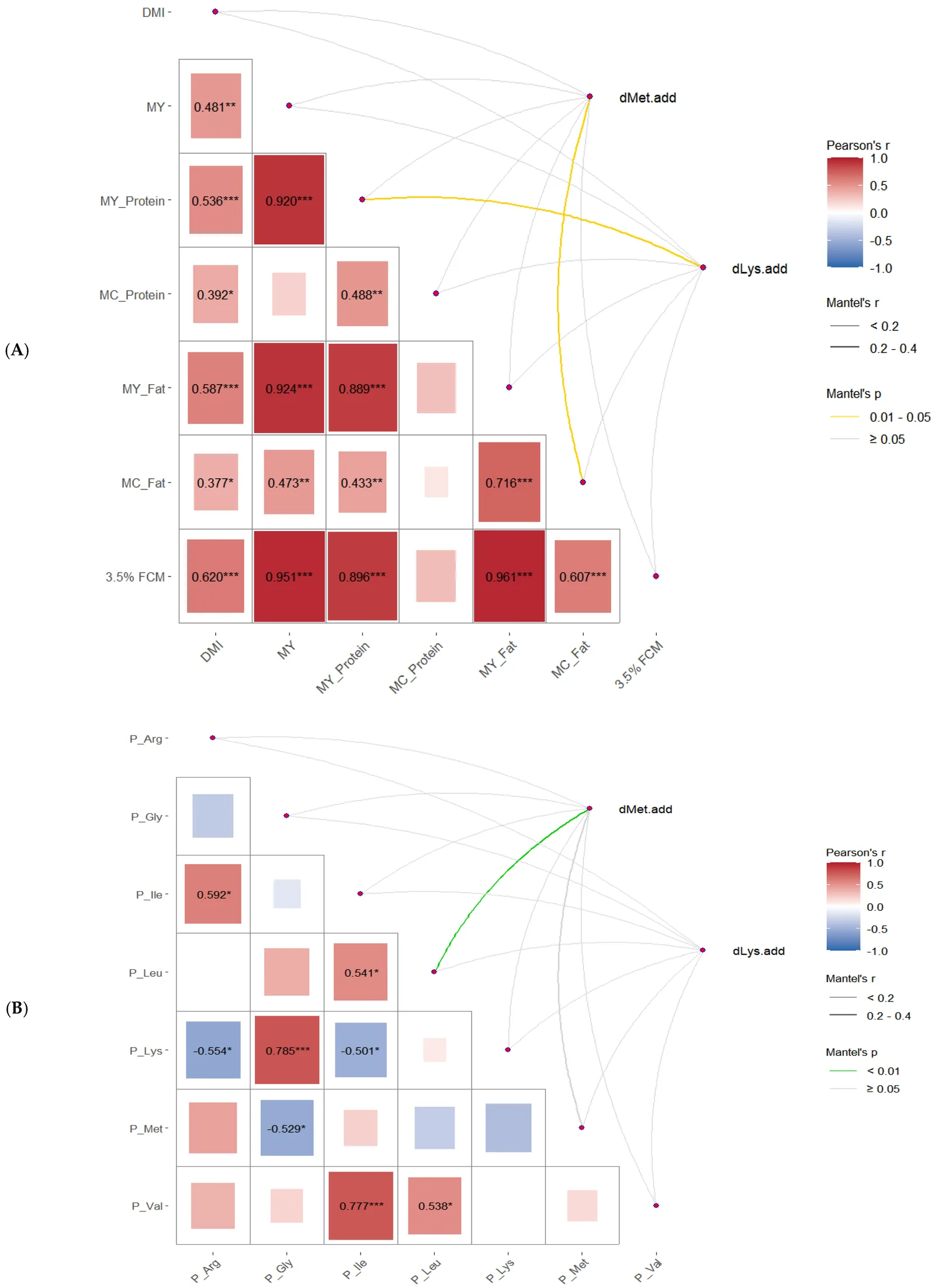
Mantel test correlation analysis among *dMet*, *dLys* and significantly affected traits of meta-analysis in Holstein cows using the Pearson correlation analysis. The colors of squares represent positive correlation (red) and negative correlation (blue) (* p < 0.05, ** p < 0.01, *** p < 0.001) among indicators. The connecting lines indicate relationships among *dMet*, *dLys* and indicators. *p*-values are represented using colors and r values using the line thickness. (**A**) Mantel test for production and lactation performance (*n* = 35). MY, milk yield (kg/d); MC, milk concentration (%). (**B**) Mantel test for plasma AA (*n* =16). *p*, plasma.

To comprehensively investigate the effect of RPML supplementation, we conducted a Mantel test analysis to examine the correlation among RPML supplementation levels and significantly affected traits in subgroup analysis. To obtain more rigorous results, we restricted the correlation analysis to Holstein-related data only. The results showed that supplementary dosage of *dMet* was significantly correlated with milk fat concentration and plasma Leu concentration (*p* < 0.01). Meanwhile, supplementary dosage of *dLys* was correlated with milk protein yield (*p* < 0.05) (Figure 2).

The correlation heatmap revealed that DMI was positively correlated with lactation performance, including yields of milk, fat, and protein, and concentrations of fat and protein. The majority of lactation parameters exhibited significantly positive inter-correlations (*p* < 0.05), with the exception that milk protein concentration was not correlated with milk yield, fat yield, fat concentration, and 3.5% FCM (*p* > 0.05) (Figure 2A). Interestingly, plasma Arg concentration was significantly positively correlated with plasma Ile concentration (r = 0.592, *p* < 0.05) and negatively correlated with plasma Lys concentration (r = −0.554, *p* < 0.05). Plasma Gly concentration was significantly positively correlated with plasma Lys concentration (r = 0.785, *p* < 0.001) and negatively correlated with plasma Met concentration (r = −0.529, *p* < 0.05). Plasma Ile concentration was positively correlated with plasma Leu concentration (r = 0.541, *p* < 0.05) and Val concentration (r = 0.777, *p* < 0.001), whilst being negatively correlated with plasma Lys concentration (r = −0.501, *p* < 0.05). Additionally, plasma Leu concentration was positively correlated with plasma Val concentration (r = 0.538, *p* < 0.05) (Figure 2B).

### 3.10. Regression Analysis of RPML Supplementation in Holstein Cows

The significantly affected traits were selected for regression analysis according to subgroup analysis results. To reduce the influence of relevant covariates and improve model precision, for each trait we selected only the correspondingly significantly affected subgroup for regression analysis. If multiple covariates influence the same trait in subgroup analysis, these covariates should be applied simultaneously for data stratification. For instance, subgroup analysis results showed that breed, parity, DIM, and silage type of diet had a significant influence on the effect sizes of RPML on milk yield. Therefore, in the response model of milk yield, we restricted the analysis to Holstein cows, multiparous cows, those in the early-lactation stage, fed corn silage and fed a high-CP diet. As shown in Table S4, a total of 11 regression models were constructed. The response of milk fat concentration to *dMet* and *dLys* is described by the following equation:

*Milk Fat Concentration* = 0.2462 − 0.0446 *dMet* + 0.0101 *dLys* + 0.0002 dMet*dLys + 0.0008 *dMet*^2^ − 0.0001 *dLys*^2^

This model was statistically significant overall (*p* < 0.01526) with an adjusted R^2^ of 0.4966, indicating that the model explained a substantial proportion of the variance. Similarly, the regression models for milk protein yield, fat yield, 3.5% FCM yield, lactose yield, DMI and plasma Met concentration were also significant (*p* < 0.05), but negative adjusted R^2^ values were observed in the models for DMI and plasma Met. Additionally, the regression models for the others were not significant (*p* > 0.05).

### 3.11. Publication Bias and Sensitivity Analysis

Egger’s tests suggested that 3.5% FCM, plasma Arg and Met from all significantly affected traits showed publication bias (*p* < 0.05). Additionally, BW and MUN from non-significantly affected parameters also indicated publication bias (*p* < 0.05) (Table 2, Table 3 and Table 4). The sensitivity analysis showed that all the estimated values were within the CIs after excluding a single datum (Figure S2). These results supported the fact that every single study did not affect the results of the meta-analysis, and our meta-analysis results were robust.

## 4. Discussion

### 4.1. The Implications of This Meta-Analysis

Dairy cows, particularly high-yielding individuals, face substantial metabolic challenges during lactation, characterized by increased demand for metabolizable protein to sustain milk synthesis [22]. Rumen-protected AA has emerged as a critical nutritional intervention because they bypass ruminal degradation and become available for absorption in the small intestine, thereby optimizing the AA profile reaching the mammary gland [23]. Met and Lys are frequently identified as the first and second limiting AAs for milk protein production in corn–soybean meal-based diets [24]. Numerous supplementation strategies with varying dosages and forms of RPML have been implemented in dairy production globally. However, the effectiveness remains inconsistent across studies due to divergent experimental conditions [12]. Therefore, this meta-analysis will systematically evaluate the effects of dietary RPML supplementation on dairy cow performance while identifying potential sources of heterogeneity. The results will enable dairy producers and nutritionists to improve rumen-protected AA application strategies, refine dietary formulations, and maximize the economic returns of milk production while reducing environmental N excretion.

### 4.2. Effects of Breed, Parity, and Lactation Stage on RPML Supplementation

Our subgroup analysis revealed that physiological responsiveness to RPML was substantially modulated by cow breed, parity, and lactation stage. Holsteins exhibited significant improvements in milk fat concentration and yields of milk, protein, and fat with RPML supplementation, whereas other breeds failed to demonstrate comparable responses in corresponding traits. This breed-specific divergence may be attributed to the distinct genetic selection histories and divergent metabolic capacities among cattle breeds. Holstein have undergone intensive selection for milk production traits for decades, resulting in elevated metabolic demands and greater nutrient requirements that predispose them to more pronounced responses to essential AA supplementation [25]. Furthermore, breed differences in ruminal fermentation characteristics, gut morphology, and intestinal AA absorption efficiency may contribute to the variable post-ruminal availability of Met and Lys, thereby influencing the efficacy of RPML supplementation [26,27].

Additionally, the yields of milk, ECM, protein, fat, and 3.5% FCM were increased in multiparous cows but not in mixed-parity cows, namely primiparous and multiparous mixed cows. This parity-dependent discrepancy in responsiveness likely reflects the substantial physiological and metabolic differences between primiparous and multiparous cows. Primiparous cows are still undergoing skeletal growth and mammary gland development during their first lactation, which competes with lactation for available nutrients and may attenuate their responsiveness to RPML [28]. Multiparous cows generally possess more developed rumen papillae, greater intestinal absorptive surface area, and higher DMI, enabling them to withstand the metabolic demands of peak lactation better and effectively channel supplemental AA toward milk synthesis [29,30].

Furthermore, the stage-of-lactation-specific patterns underscore the dynamic nature of nutrient partitioning throughout the lactation cycle. During the transition period, cows experience dramatic shifts in hormonal profiles and metabolic states [31]. The supplementation of RPML improved the health status and immune responses of cows and reduced the incidence rate of metabolic diseases [32]. As lactation advances, the progressive shift in nutrient partitioning priorities, namely directing nutrients toward body tissue replenishment rather than exclusive milk synthesis, may explain the attenuated responsiveness in fat-related traits, as milk fat synthesis becomes more dependent on energy status and acetate availability rather than AA supply alone [33].

### 4.3. Effects of Dietary Silage Type and CP Level on RPML Supplementation

Our subgroup analysis revealed distinct patterns of response to RPML supplementation depending on basal silage type and dietary CP level. Cows fed corn silage-based diets supplied RPML exhibited significant improvements in milk yield and protein concentration. In contrast, RPML supplementation to grass silage-based diets significantly influenced milk fat yield, 3.5% FCM, ECM, and casein N concentration. This divergence may be attributed to the fundamentally different nutritional profiles and ruminal fermentation characteristics between these two forage sources [34]. Corn silage typically contains higher concentrations of non-fiber carbohydrates and readily fermentable carbohydrates compared to grass silage, which promote greater ruminal propionate production and microbial protein synthesis [35]. The enhanced energy availability in corn silage-based diets creates a more favorable environment for the efficient utilization of absorbed AAs, allowing RPML to effectively correct the limiting AA deficiency and stimulate milk protein synthesis [36]. In contrast, grass silage-based diets are generally characterized by higher CP content and elevated rumen-degradable protein levels but lower energy density [34]. The imbalance in energy-to-protein ratio in grass silage may result in excessive ruminal ammonia production that exceeds microbial capture capacity, leading to inefficient N utilization and diminished responsiveness of milk protein concentration to RPML [37]. Additionally, RPML supplementation to grass silage-based diets significantly influences fat-related traits, potentially through modifications in ruminal bio-hydrogenation pathways and alterations in the supply of specific fatty acid precursors [38].

Additionally, the differential response to RPML based on dietary CP level provides additional insights into the conditional efficacy of AA supplementation. Milk yield, fat yield, fat concentration, and 3.5% FCM were significantly enhanced by RPML only under a high-CP diet. In the high-CP diet, the elevated ruminal ammonia pool supports robust microbial protein synthesis, and the addition of RPML optimizes the post-ruminal AA profile, thereby promoting the efficiency of metabolizable protein utilization for milk synthesis [39]. The mechanism exploration showed that RPML under high-CP conditions facilitates favorable nutrient partitioning, potentially through enhancing insulin signaling that directs nutrients toward mammary gland metabolism [5]. Additionally, in high-CP diets, abundant nitrogen substrate is available; when amino acid profile is imbalanced, excess nitrogen is excreted. On the other hand, in low-CP diets, protein supply is limiting, and supplemental amino acids mainly fill limiting amino acid gaps, yet total nitrogen intake remains constrained, so the scope for further response is relatively limited. Supplemental amino acids can optimize amino acid pattern and redirect more absorbed nitrogen toward tissue deposition instead of urea excretion, thus producing a larger magnitude of improvement in N-use efficiency. Moreover, the plasma concentrations of Ala, Asn, Gly, Leu were significantly affected by RPML only under a low-CP diet, not in a high-CP diet. The underlying mechanism warrants further investigation.

### 4.4. The Combining Effects of Met and Lys Supplementation in Dairy Cow

The joint supplementation of Met and Lys in high-yielding dairy cows represents a critical precision nutrition strategy that transcends the limitations of individual AA fortification. Single AA supplementation often fails to achieve full physiological efficacy due to relative deficiencies in complementary AAs, resulting in imbalanced absorbable profiles, enhanced catabolic losses, and diminished N utilization efficiency [40]. Combined supplementation optimizes the intestinal AA supply to approximate the ideal pattern for milk protein synthesis. This holistic optimization maximizes mammary uptake efficiency, yielding supra-additive improvements in milk protein output that exceed the arithmetic sum of individual effects, while concomitantly reducing urinary N excretion [41]. Furthermore, their integrated roles in antioxidant defense, immune competence, and hepatic lipid metabolism sustain overall metabolic homeostasis, thereby extending peak lactation persistency [32].

The present study revealed that the regression models were statistically significant overall and exhibited high explanatory power (adjusted R^2^), but most of the coefficients did not reach statistical significance. This phenomenon is not uncommon in regression analysis and can be attributed to multi-collinearity among polynomial terms, insufficient sample size, non-orthogonal data, etc. [42]. All these results indicated a potential combining effect of Met and Lys on milk production. Still, their independent effects and optimal dosage and ratio could not be determined, resulting in limited predicted capacity of the models and insufficient practical supplementation strategies.

### 4.5. The Limitations of This Meta-Analysis

This meta-analysis has several limitations deserving further mention. First, heterogeneity was prevalent, and the values were relatively high. In the significantly affected parameters, most heterogeneity was greater than 50%, indicating high heterogeneity. This may be attributed to substantial variations in experimental conditions, such as body weight, milk yield, supplemental dosages, etc. Second, caution should be exercised when interpreting the subgroup analysis results, given the marked imbalance in sample sizes and small trails across subgroups. These findings should be interpreted as exploratory and require confirmation in larger datasets. Third, the limited number of studies and data included in the correlation analysis may compromise the generalizability and reliability of the findings. Fourth, regression models exhibited low or even negative adjusted R^2^ values, with significant overall model effects but non-significant individual predictors. Therefore, the regression models should be interpreted as exploratory rather than predictive. Multicollinearity among variables and limited sample size may have compromised the stability of parameter estimates. Future work with larger datasets and careful variable screening is needed to validate these relationships and build robust predictive models. Nevertheless, these findings may provide valuable insights for future investigations on RPML supplementation strategies.

## 5. Conclusions

Our meta-analysis indicated that RPML supplementation significantly enhanced milk yield (0.500 kg/d), ECM (1.378 kg/d), protein yield (0.038 kg/d), fat yield (0.030 kg/d), protein concentration (0.052%), fat concentration (0.043%), milk total N (0.010%), and casein N (0.007%) in dairy cows. Plasma concentrations of Arg, Asn, Ile, Leu, Lys and Met increased markedly, while Gly decreased. Subgroup and correlation analyses identified breed, parity, lactation stage, basal diet, and CP level as key moderators. Supplementary dosage of *dMet* was correlated with milk fat concentration and plasma Leu, while *dLys* dosage was correlated with milk protein yield. The overall response model significance suggests a signal of combining effects between Met and Lys. More precise and targeted future research will be essential to effectively improve the utilization of RPML in dairy cow production and thereby enhance overall cattle performance.

## Figures and Tables

**Table 1:** Summary statistics for variables used in this meta-analysis.

	Treatment					Control				
Item	*n*	Mean	SD	Maximum	Minimum	*n*2	Mean2	SD2	Maximum2	Minimum2
BW, kg	47	631.58	68.21	814.00	417.00	47	630.00	70.38	845.00	416.00
DMI, kg/d	71	20.85	4.23	30.00	9.40	71	20.61	4.29	29.40	9.30
BCS	31	3.04	0.38	3.83	2.20	31	3.00	0.36	3.86	2.20
Milk Yield, kg/d	74	32.99	6.97	46.90	18.80	74	32.14	6.53	47.05	18.80
ECM, kg/d	31	38.90	6.22	50.20	28.70	31	37.40	5.85	48.10	25.50
Milk Protein, kg/d	73	1.05	0.21	1.84	0.64	73	1.00	0.19	1.84	0.62
Milk Protein, %	68	3.22	0.27	4.18	2.62	68	3.16	0.25	3.76	2.67
Milk Fat, kg/d	75	1.25	0.27	1.88	0.77	75	1.21	0.25	1.85	0.75
Milk Fat, %	72	3.80	0.41	5.15	3.13	72	3.77	0.40	4.96	3.07
3.5%FCM, kg/d	51	34.15	7.45	51.00	24.158	51	32.86	6.66	49.00	24.57
Milk Lactose, kg/d	31	1.69	0.34	2.33	1.029	31	1.66	0.32	2.34	0.99
Milk Lactose, %	37	4.81	0.13	5.10	4.55	37	4.80	0.13	5.06	4.43
MUN, mg/dL	26	11.84	2.61	18.30	7.00	26	12.34	3.31	18.30	6.90
Total N, %	11	0.56	0.05	0.66	0.488	11	0.55	0.03	0.59	0.491
Casein N, %	11	0.41	0.05	0.51	0.3296	11	0.40	0.04	0.44	0.312
Whey N, %	11	0.11	0.01	0.1312	0.091	11	0.11	0.01	0.136	0.091
Ala, μmol/L	27	235.60	61.43	444.70	143.20	27	221.70	42.05	282.00	139.90
Arg, μmol/L	26	76.16	34.40	133.00	3.90	26	70.80	31.93	134.00	9.00
Asn, μmol/L	16	40.68	22.20	99.00	23.00	16	37.97	19.08	82.20	21.30
Asp, μmol/L	25	33.11	41.39	142.10	5.00	25	32.27	39.66	131.30	5.00
Gln, μmol/L	16	205.68	40.91	288.70	148.60	16	204.85	44.63	282.90	147.50
Glu, μmol/L	27	128.94	145.12	665.30	24.00	27	121.43	124.03	462.70	23.00
Gly, μmol/L	27	316.07	98.64	656.80	176.10	27	321.30	84.97	534.00	183.60
His, μmol/L	26	47.68	23.13	146.70	19.40	26	47.54	21.02	133.90	24.20
Ile, μmol/L	30	96.06	23.79	158.90	46.30	30	91.31	22.56	163.90	56.80
Leu, μmol/L	31	152.96	75.30	408.20	67.40	31	145.62	68.69	367.60	77.90
Lys, μmol/L	31	70.40	23.13	121.10	27.00	31	58.47	18.46	106.80	25.00
Met, μmol/L	31	26.37	10.38	49.00	7.50	31	17.93	6.56	30.60	7.00
Phe, μmol/L	30	38.56	12.56	62.00	18.00	30	38.22	11.88	62.00	20.40
Pro, μmol/L	24	84.69	24.46	164.20	46.00	24	83.20	21.77	146.00	45.00
Ser, μmol/L	27	65.33	36.66	138.20	3.40	27	66.26	36.31	115.10	3.30
Thr, μmol/L	31	78.56	31.00	149.70	20.60	31	79.31	29.75	140.00	24.30
Trp, μmol/L	13	39.69	14.56	79.00	25.00	13	38.99	14.84	80.70	25.00
Tyr, μmol/L	26	35.73	17.78	69.90	7.30	26	35.45	16.55	70.90	8.30
Val, μmol/L	31	214.90	51.85	382.80	117.50	31	208.44	51.72	394.40	96.00

Ala: Alanine; Arg: Arginine; Asn: Asparagine; Asp: Aspartic acid; BW: body weight; BCS: body condition score; DMI: dry matter intake; ECM: energy-corrected milk, FCM: fat-corrected milk; Gln: Glutamine; Glu: Glutamic acid; Gly: Glycine; His: Histidine; Ile: Isoleucine; Leu: Leucine; Lys: Lysine; Met: Methionine; MUN; milk urea nitrogen; *n*: number of effect size; N: nitrogen; Phe: Phenylalanine; Pro: Proline; Ser: Serine; Thr: Threonine; Trp: Tryptophan; Tyr: Tyrosine; Val: Valine.

**Table 2:** The meta-analysis on the effects of RPML on the body performance of dairy cows.

		RMD (95% CI)				Heterogeneity		Egger’s Test
	*n*	Control Mean	Random Effect	[95% CI]	*p*-Value	*p*-Value	I^2^ (%)	*p*-Value
BW, kg	47	630.00	0.730	−2.414 3.875	0.649	0.001	44.20	0.01
DMI, kg/d	71	20.61	0.283	−0.039 0.605	0.085	0	81.50	0.20
BCS	31	3.00	0.044	−0.017 0.106	0.159	0	83.00	0.588

BW: body weight; BCS: body condition score; CI: confidence interval; DMI: dry matter intake; *n*: number of effect size; RMD: raw mean difference.

**Table 3:** The meta-analysis on the effects of RPML on the lactation performance of dairy cows.

		RMD (95% CI)				Heterogeneity		Egger’s Test
	*n*	Control Mean	Random Effect	[95% CI]	*p*-Value	*p*-Value	I2 (%)	*p*-Value
Milk Yield, kg/d	74	32.14	0.500	0.210 0.791	0.001	0	62.90	0.581
ECM, kg/d	31	37.40	1.378	0.457 2.299	0.003	0	64.70	0.189
Milk Protein, kg/d	73	1.00	0.038	0.025 0.05	0	0	69.00	0.058
Milk Protein, %	68	3.16	0.052	0.035 0.07	0	0	59.10	0.457
Milk Fat, kg/d	75	1.21	0.030	0.011 0.049	0.002	0	75.80	0.169
Milk Fat, %	72	3.77	0.043	0.011 0.076	0.009	0.006	32.30	0.105
3.5% FCM, kg/d	51	32.86	0.778	0.265 1.291	0.003	0	67.40	0.001
Milk Lactose, kg/d	31	1.66	0.030	−0.005 0.065	0.090	0	72.50	0.474
Milk Lactose, %	37	4.80	0.002	−0.015 0.019	0.822	0.007	40.20	0.552
MUN, mg/dL	26	12.34	−0.451	−1.207 0.306	0.243	0	92.40	0.009
Total N, %	11	0.55	0.010	0.002 0.017	0.009	0.007	58.70	0.185
Casein N, %	11	0.40	0.007	0 0.013	0.045	0.045	46.30	0.241
Whey N, %	11	0.11	0.002	0 0.005	0.081	0.949	0.00	0.557

CI: confidence interval; ECM: energy-corrected milk, FCM: fat-corrected milk; MUN; milk urea nitrogen; *n*: number of effect size; N: nitrogen; RMD: raw mean difference.

**Table 4:** The meta-analysis on the effects of RPML on the plasma AA of dairy cows.

		RMD (95% CI)				Heterogeneity		Egger’s Test
	*n*	Control Mean	Random Effect	[95% CI]	*p*-Value	*p*-Value	I2 (%)	*p*-Value
Ala, μmol/L	27	221.70	5.236	−0.372 10.844	0.067	0.645	0	0.446
Arg, μmol/L	26	70.80	4.071	1.61 6.531	0.001	0.007	45.20	0.025
Asn, μmol/L	16	37.97	1.693	0.333 3.053	0.015	0.588	0	0.345
Asp, μmol/L	25	32.27	0.011	−0.334 0.356	0.951	0.593	0	0.330
Gln, μmol/L	16	204.85	1.370	−4.486 7.227	0.647	0.647	0	0.796
Glu, μmol/L	27	121.46	0.797	−0.364 1.957	0.178	0.537	0	0.213
Gly, μmol/L	27	321.31	−14.408	−24.764 −4.051	0.006	0.040	34.90	0.751
His, μmol/L	26	47.54	−0.254	−1.613 1.105	0.714	0.270	13.40	0.812
Ile, μmol/L	30	91.31	5.641	3.017 8.264	0	0.422	2.80	0.077
Leu, μmol/L	31	145.62	5.325	1.438 9.212	0.007	0.239	14.50	0.200
Lys, μmol/L	31	58.47	10.112	7.119 13.106	0	0	58.60	0.152
Met, μmol/L	31	17.93	8.242	6.139 10.345	0	0	88.80	0.012
Phe, μmol/L	30	38.22	0.669	−0.377 1.714	0.210	0.017	38.90	0.313
Pro, μmol/L	24	83.21	1.323	−1.422 4.096	0.345	0.053	34.20	0.622
Ser, μmol/L	27	66.26	0.091	−1.179 1.36	0.889	0.095	27.40	0.074
Thr, μmol/L	31	79.31	−0.618	−2.897 1.661	0.595	0.197	17.40	0.699
Trp, μmol/L	13	38.99	0.564	−1.014 2.142	0.484	0.785	0	0.840
Tyr, μmol/L	26	35.45	0.482	−1.051 2.016	0.538	0.221	16.90	0.778
Val, μmol/L	31	208.44	7.072	−1.244 15.387	0.096	0	58.80	0.273

Ala: Alanine; Arg: Arginine; Asn: Asparagine; Asp: Aspartic acid; Gln: Glutamine; Glu: Glutamic acid; Gly: Glycine; His: Histidine; Ile: Isoleucine; Leu: Leucine; Lys: Lysine; Met: Methionine; *n*: number of effect size; Phe: Phenylalanine; Pro: Proline; Ser: Serine; Thr: Threonine; Trp: Tryptophan; Tyr: Tyrosine; Val: Valine.

## Data Availability

The datasets presented in this study are available on request from the corresponding author upon reasonable request.

## Supplementary Materials

The following supporting information can be downloaded at: https://www.mdpi.com/article/10.3390/ani16213442/s1. Table S1 The sub-group analysis on the effects of RPML on DMI of dairy cows; Table S2 The sub-group analysis on the effects of RPML on the lactation performance of dairy cows; Table S3 The sub-group analysis on the effects of RPML on the plasma AA of dairy cows; Table S4 Parameters of the models between supplementary RPML and performance of dairy cows; Figure S1. The flowchart of the search and selection strategies of eligible studies; Figure S2. Sensitive analysis results.

## Abbreviations

The following abbreviations are used in this manuscript:

AA	Amino acid
BCS	Body condition score
BW	Body weight
CI	Confidence intervals
DIM	Days in milk
*dMet*	Digestible Met
DMI	Dry matter intake
*dLys*	Digestible Lys
ECM	Energy-corrected milk
FCM	Fat-corrected milk
MUN	Milk urea nitrogen
N	Nitrogen
PRISMA	Preferred reporting items for systematic reviews and meta-analyses
RMD	Raw mean difference
RPML	Rumen-protected Met and Lys
