# Supplementary material for: Effect of Dietary Rumen-Protected Methionine and Lysine on Performance and Plasma Amino Acid of Dairy Cows: A Meta-Analysis
Source: Animals (Basel). 2026 Oct 31;16(21):3442. doi: 10.3390/ani16213442 (PMC13761621; doi:10.3390/ani16213442)
Supplement: Supplementary file 1 [file animals-16-03442-s001.zip › 2 Table S1,S2,S3,S4-20260927-1019-1028.pdf]

Table S1 The sub-group analysis on the effects of RPML on DMI of dairy cows

		RMD (95% CI)					Heterogeneity	
Item	n	Control mean	Random effect	95% CI		P-value	P-value	I^2 (%)
Breed								
Holstein	59	21.06	0.378	0.023	0.734	0.037	0	82.80
others	12	18.39	-0.379	-0.681	-0.076	0.014	0.998	0
Total	71	20.61	0.283	-0.039	0.605	0.085	0	81.50
Parity								
multiparous	48	20.68	0.408	-0.052	0.867	0.082	0	85.50
mix	22	20.405	-0.088	-0.32	0.143	0.454	0.947	0
Total	70	20.59	0.276	-0.062	0.614	0.109	0	81.80
DIM								
pre	22	18.02	0.576	0.102	1.051	0.017	0	59.90
early	37	21.67	0.150	-0.416	0.716	0.605	0	88.10
mid	12	22.09	0.064	-0.195	0.324	0.627	0.866	0
Total	71	20.61	0.283	-0.039	0.605	0.085	0	81.50
basic feed								
corn silage	47	20.97	0.259	-0.192	0.709	0.26	0	84.90
grass silage	11	18.25	0.522	-0.39	1.434	0.262	0.014	54.80
mix	13	21.32	0.151	-0.077	0.379	0.194	0.531	0
Total	71	20.61	0.283	-0.039	0.605	0.085	0	81.50
CP								
low	25	20.544	0.055	-0.137	0.247	0.576	0.990	0
high	46	20.65	0.386	-0.116	0.887	0.132	0	86.60
Total	71	20.61	0.283	-0.039	0.605	0.085	0	81.50

Table S2 The sub-group analysis on the effects of RPML on the lactation performance of dairy cows

Item	n	RMD (95% CI)					Heterogeneity	
		Control mean	Random effect	[95% CI]		P-value	P-value	I^2 (%)
**Milk Yield, kg/d**								
breed								
Holstein	61	33.63	0.658	0.325	0.992	<0.001	0	64.50
others	13	25.17	-0.136	-0.628	0.357	0.589	0.145	29.90
Total	74	32.14	0.500	0.210	0.791	0.001	0	62.90
parity								
multiparous	53	32.25	0.552	0.226	0.878	0.001	0	64.80
mix	21	31.86	0.340	-0.324	1.004	0.315	0.001	55.30
Total	74	32.14	0.500	0.210	0.791	0.001	0	62.90
DIM								
pre	18	36.925	1.268	0.362	2.173	0.006	0.156	25.40
early	43	30.62	0.670	0.296	1.044	<0.001	0	66.40
mid	13	30.55	-0.179	-0.597	0.240	0.402	0.026	48.20
Total	74	32.14	0.500	0.210	0.791	0.001	0	62.90
basic feed								
corn silage	46	31.8	0.486	0.101	0.871	0.013	0	71.80
grass silage	13	29.32	0.729	-0.034	1.493	0.061	0.016	51.60
mix	15	35.64	0.527	0.084	0.969	0.020	0.711	0
Total	74	32.14	0.500	0.210	0.791	0.001	0	62.90
CP								
low	28	31.85	0.134	-0.228	0.495	0.469	0	62.30
high	46	32.32	0.952	0.465	1.439	<0.001	0	64.00
Total	74	32.14	0.500	0.210	0.791	0.001	0	62.90
**ECM, kg/d**								
parity								
multiparous	18	37.79	2.418	1.056	3.779	0.001	0	77.40
mix	13	36.85	-0.034	-1.003	0.935	0.945	0.995	0
Total	31	37.4	1.378	0.457	2.299	0.003	0	64.70
DIM								
pre	15	38.17	1.882	0.792	2.972	0.001	0.23	20.00
early	14	35.81	0.992	-0.645	2.629	0.235	0	79.40
mid								
Total	29	37.03	1.450	0.416	2.484	0.006	0	66.40
basic feed								
corn silage	17	38.18	1.233	-0.145	2.611	0.079	0	75.40
grass silage	7	33.16	3.667	2.102	5.231	0	0.603	0
mix	7	39.73	0.087	-0.862	1.037	0.857	0.994	0
Total	31	37.4	1.378	0.457	2.299	0.003	0	64.70

**Milk Protein, kg/d**								
breed								
Holstein	62	1.04	0.045	0.030	0.059	＜0.001	0	70.00
others	11	0.81	0.008	-0.009	0.024	0.352	0.16	30.10
Total	73	1	0.038	0.025	0.050	＜0.001	0	69.00
parity								
multiparous	49	1	0.047	0.032	0.062	＜0.001	0	74.10
mix	23	1.01	0.012	-0.007	0.031	0.231	0.054	34.40
Total	72	1	0.038	0.025	0.050	＜0.001	0	69.40
DIM								
pre	16	1.0845	0.065	0.035	0.095	＜0.001	0.182	24.00
early	45	0.979	0.045	0.029	0.062	＜0.001	0	72.60
mid	12	0.9775	0.001	-0.010	0.012	0.851	0.393	5.40
Total	73	1.002	0.038	0.025	0.050	＜0.001	0	69.00
basic feed								
corn silage	43	0.99	0.037	0.019	0.054	＜0.001	0	78.10
grass silage	14	0.93	0.040	0.018	0.063	＜0.001	0.033	45.50
mix	16	1.11	0.031	0.010	0.051	0.003	0.376	6.80
Total	73	1	0.038	0.025	0.050	＜0.001	0	69.00
CP								
low	27	0.97	0.029	0.012	0.045	0.001	0	69.70
high	46	1.02	0.047	0.029	0.066	＜0.001	0	69.10
Total	73	1	0.038	0.025	0.050	＜0.001	0	69.00
**Milk Protein, %**								
breed								
Holstein	60	3.11	0.049	0.032	0.067	＜0.001	0	51.90
others	8	3.52	0.076	0.005	0.147	0.035	0	80.60
Total	68	3.16	0.052	0.035	0.070	＜0.001	0	59.10
parity								
multiparous	46	3.12	0.055	0.036	0.073	＜0.001	0	53.80
mix	21	3.26	0.053	0.008	0.097	0.020	0	69.50
Total	67	3.16	0.053	0.035	0.070	＜0.001	0	59.70
DIM								
pre	16	3.04	0.040	-0.013	0.093	0.136	0	73.30
early	40	3.19	0.065	0.043	0.088	＜0.001	0	49.90
mid	12	3.22	0.035	0.011	0.059	0.004	0.044	45.20
Total	68	3.16	0.052	0.035	0.070	＜0.001	0	59.10
basic feed								
corn silage	41	3.19	0.060	0.037	0.082	＜0.001	0	55.30
grass silage	11	3.11	0.023	-0.04	0.087	0.473	0	81.70
mix	16	3.13	0.034	0.017	0.052	＜0.001	0.44	1.00
Total	68	3.16	0.052	0.035	0.070	＜0.001	0	59.10
CP								
low	24	3.1275	0.051	0.030	0.072	＜0.001	0.195	19.50

high	44	3.18	0.050	0.027	0.074	<0.001	0	68.20
Total	68	3.16	0.052	0.035	0.070	<0.001	0	59.10
**Milk Fat, kg/d**								
breed								
Holstein	62	1.24	0.034	0.012	0.055	0.002	0	79.00
others	13	1.05	0.006	-0.015	0.027	0.563	0.996	0
Total	75	1.21	0.030	0.011	0.049	0.002	0	75.80
parity								
multiparous	56	1.18	0.038	0.016	0.059	0.001	0	80.70
mix	18	1.3	-0.007	-0.040	0.025	0.655	0.896	0
Total	74	1.211	0.029	0.010	0.048	0.003	0	76.00
DIM								
pre	18	1.37	0.075	0.038	0.112	<0.001	0.36	7.90
early	45	1.17	0.023	0	0.046	0.049	0	57.20
mid	12	1.1315	0.012	-0.030	0.054	0.576	0	93.50
Total	75	1.21	0.030	0.011	0.049	0.002	0	75.80
basic feed								
corn silage	46	1.22	0.019	-0.002	0.039	0.079	0	65.00
grass silage	14	1.0475	0.045	0.009	0.080	0.014	0.051	41.80
mix	15	1.35	0.040	-0.006	0.086	0.089	0.001	60.00
Total	75	1.21	0.030	0.011	0.049	0.002	0	75.80
CP								
low	26	1.18	0.009	-0.010	0.028	0.349	0	56.20
high	49	1.23	0.047	0.020	0.075	0.001	0	71.30
Total	75	1.21	0.030	0.011	0.049	0.002	0	75.80
**Milk Fat, %**								
breed								
Holstein	64	3.71	0.039	0.005	0.074	0.025	0.013	30.30
others	8	4.25625	0.072	-0.039	0.182	0.202	0.045	51.30
Total	72	3.77	0.043	0.011	0.076	0.009	0.006	32.30
parity								
multiparous	48	3.68	0.034	-0.003	0.071	0.069	0.057	25.60
mix	23	3.96	0.045	-0.029	0.120	0.232	0.027	39.70
Total	71	3.77	0.040	0.006	0.074	0.021	0.008	31.20
DIM								
pre	18	3.71	0.061	-0.005	0.127	0.07	0.9	0
early	41	3.81	0.020	-0.032	0.072	0.455	0	51.00
mid	13	3.73	0.039	-0.006	0.084	0.089	0.417	3.00
Total	72	3.77	0.043	0.011	0.076	0.009	0.006	32.30
basic feed								
corn silage	44	3.83	0.032	-0.018	0.082	0.215	0	51.20
grass silage	11	3.49	0.053	-0.010	0.115	0.098	0.73	0
mix	17	3.81	0.052	0.012	0.091	0.010	0.881	0
Total	72	3.77	0.043	0.011	0.076	0.009	0.006	32.30

CP								
low	27	3.76	0.016	-0.023	0.056	0.416	0.559	0
high	45	3.78	0.063	0.017	0.109	0.007	0.002	41.40
Total	72	3.77	0.043	0.011	0.076	0.009	0.006	32.30
**3.5% FCM, kg/d**								
parity								
multiparous	41	33.89	0.848	0.282	1.415	0.003	0	67.20
mix	9	28.74	0.394	-0.971	1.760	0.572	0	74.00
Total	50	32.94	0.768	0.251	1.284	0.004	0	67.90
DIM								
pre	14	38.69	2.659	1.597	3.721	＜0.001	0.454	0
early	26	31.48	0.764	-0.113	1.641	0.088	0	73.00
mid	11	28.7	-0.022	-0.546	0.501	0.933	0.013	55.60
Total	51	32.86	0.778	0.265	1.291	0.003	0	67.40
basic feed								
corn silage	35	32.07	0.491	-0.073	1.054	0.088	0	71.10
grass silage	8	33.85	4.083	2.489	5.676	＜0.001	0.772	0
mix	8	35.3375	0.423	-0.553	1.400	0.396	0.316	14.50
Total	51	32.86	0.778	0.265	1.291	0.003	0	67.40
CP								
low	20	31.03	-0.129	-0.538	0.279	0.535	0.183	22.00
high	31	34.04	1.589	0.718	2.461	＜0.001	0	73.30
Total	51	32.86	0.778	0.265	1.291	0.003	0	67.40
**Milk Lactose, kg/d**								
parity								
multiparous	18	1.629	0.043	-0.003	0.089	0.065	0	83.80
mix	12	1.71	0.008	-0.040	0.056	0.746	0.965	0
Total	30	1.66	0.030	-0.005	0.065	0.091	0	73.40
DIM								
pre	6	1.92	-0.013	-0.087	0.062	0.743	0.99	0
early	21	1.58	0.033	-0.009	0.075	0.124	0	80.90
mid								
Total	27	1.65	0.026	-0.011	0.064	0.169	0	75.40
basic feed								
corn silage	19	1.66	0.058	-0.001	0.116	0.053	0	80.00
grass silage								
mix	9	1.72	0.021	-0.027	0.070	0.383	0.853	0
Total	28	1.68	0.042	-0.002	0.086	0.063	0	71.30
CP								
low	9	1.65	-0.001	-0.032	0.029	0.930	0.168	31.20
high	22	1.66	0.047	-0.007	0.101	0.087	0	77.80
Total	31	1.657	0.030	-0.005	0.065	0.090	0	72.50
**Total N, %**								
basic feed								

corn silage	6	0.56	0.011	0	0.022	0.042	0.001	76.40
grass silage	5	0.5402	0.011	0.003	0.019	0.005	0.835	0
mix								
Total	11	0.55	0.010	0.002	0.017	0.009	0.007	58.70
**Casein N, %**								
breed								
Holstein	6	0.38	0.003	-0.002	0.007	0.249	0.437	0
others	5	0.4162	0.013	-0.001	0.028	0.072	0.042	59.60
Total	11	0.395	0.007	0	0.013	0.045	0.045	46.30
basic feed								
corn silage	6	0.385	0.007	-0.003	0.016	0.172	0.011	66.10
grass silage	5	0.4068	0.010	0.002	0.017	0.013	0.718	0
mix								
Total	11	0.395	0.007	0	0.013	0.045	0.045	46.30
**Whey N, %**								
breed								
Holstein	6	0.11	0.003	0	0.006	0.047	0.747	0
others	5	0.1	0	-0.004	0.005	0.856	0.988	0
Total	11	0.105	0.002	0	0.005	0.081	0.949	0
basic feed								
corn silage	6	0.108	0.003	0	0.006	0.050	0.733	0
grass silage	5	0.1018	0	-0.004	0.005	0.847	0.988	0
mix								
Total	11	0.105	0.002	0	0.005	0.081	0.949	0

Table S3 The sub-group analysis on the effects of RPML on the plasma AA of dairy cows

			RMD (95% CI)			Heterogeneity	
Item	n	Control mean	Random effect	[95% CI]	P-value	P-value	I^2 (%)
**Ala, μmol/L**							
breed							
Holstein	22	221.57	4.180	-2.176 10.537	0.197	0.703	0
others	5	222.28	8.689	-5.051 22.428	0.215	0.27	22.70
Total	27	221.7	5.236	-0.372 10.844	0.067	0.645	0
parity							
multiparous	15	228.37	0.921	-6.905 8.747	0.818	0.539	0
mix	12	213.37	9.788	1.749 17.827	0.017	0.754	0
Total	27	221.7	5.236	-0.372 10.844	0.067	0.645	0
DIM							
pre	7	199.33	8.951	-2.942 20.844	0.140	0.745	0
early	13	210.79	2.262	-6.979 11.502	0.631	0.182	26.00
mid	7	264.33	7.94	-2.986 18.865	0.154	0.927	0
Total	27	221.7	5.236	-0.372 10.844	0.067	0.645	0
basic feed							
corn silage	19	228.15	4.786	-1.842 11.415	0.157	0.435	1.70
grass silage							
mix	6	217.32	6.313	-4.497 17.123	0.252	0.733	0
Total	25	225.548	5.193	-0.416 10.802	0.070	0.629	0
CP							
low	10	245.18	10.056	0.689 19.424	0.035	0.998	0
high	17	207.89	2.695	-5.338 10.729	0.511	0.227	19.40
Total	27	221.7	5.236	-0.372 10.844	0.067	0.645	0
**Arg, μmol/L**							
breed							
Holstein	21	65.68	3.637	1.280 5.994	0.002	0.032	39.70
others	5	92.3	6.625	-6.375 19.625	0.318	0.023	64.60
Total	26	70.8	4.071	1.610 6.531	0.001	0.007	45.20
parity							
multiparous	13	68.58	2.820	-0.558 6.199	0.102	0.007	55.90
mix	13	73.02	5.772	2.773 8.771	$<$0.001	0.377	6.90
Total	26	70.8	4.071	1.610 6.531	0.001	0.007	45.20
DIM							
pre	7	41.63	1.332	-2.922 5.587	0.539	0.008	65.40
early	17	82.07	5.910	3.445 8.375	$<$0.001	0.448	0.50
mid							
Total	24	70.275	4.111	1.450 6.772	0.002	0.004	48.60
basic feed							0

corn silage	17	81.14	6.065	3.430	8.700	＜0.001	0.336	10.10
grass silage								
mix	7	63.36	4.526	1.402	7.649	0.005	0.998	0
Total	24	75.95	5.465	3.564	7.366	＜0.001	0.712	0
CP								
low	6	62.6	4.601	1.007	8.194	0.012	0.918	0
high	20	73.26	4.238	1.069	7.407	0.009	0.001	56.00
Total	26	70.8	4.071	1.610	6.531	0.001	0.007	45.20
**Asn, µmol/L**								
parity								
multiparous	8	32.25	0.434	-1.563	2.431	0.670	0.786	0
mix	8	43.6875	2.783	0.925	4.641	0.003	0.495	0
Total	16	37.97	1.693	0.333	3.053	0.015	0.588	0
DIM								
pre	5	33.1	2.834	0.534	5.135	0.016	0.826	0
early	9	39.9	1.089	-0.863	3.042	0.274	0.567	0
mid								
Total	14	37.47	1.820	0.331	3.309	0.017	0.733	0
basic feed								0
corn silage	10	40.49	1.780	-0.212	3.773	0.080	0.619	0
grass silage								
mix	6	33.77	1.622	-0.452	3.695	0.125	0.307	16.60
Total	16	37.97	1.693	0.333	3.053	0.015	0.588	0
CP								
low	5	30.88	2.836	0.685	4.987	0.010	0.699	0
high	11	41.19	0.931	-0.825	2.387	0.299	0.516	0
Total	16	37.97	1.693	0.333	3.053	0.015	0.588	0
**Gly, µmol/L**								
breed								
Holstein	22	314.36	-14.644	-26.715	-2.573	0.017	0.016	43.40
others	5	351.84	-11.937	-33.179	9.306	0.271	0.593	0
Total	27	321.31	-14.408	-24.764	-4.051	0.006	0.04	34.90
parity								
multiparous	15	331.09	-25.419	-37.746	-13.092	＜0.001	0.184	24.40
mix	12	309.075	3.170	-9.271	15.610	0.618	0.49	0
Total	27	321.31	-14.408	-24.764	-4.051	0.006	0.04	34.90
DIM								
pre	7	364.29	-24.638	-73.990	24.713	0.328	0.025	58.60
early	13	292.04	-5.506	-18.261	7.248	0.397	0.258	18.40
mid	7	332.67	-18.428	-30.668	-6.189	0.003	0.47	0
Total	27	321.31	-14.408	-24.764	-4.051	0.006	0.04	34.90
basic feed								
corn silage	19	329.42	-19.415	-31.369	-7.461	0.001	0.045	38.50
grass silage								0

mix	6	289.95	-1.788	-17.639	14.063	0.825	0.544	0
Total	25	319.944	-14.572	-24.650	-4.494	0.005	0.055	33.30
CP								
low	10	323.9	-17.743	-33.430	-2.056	0.027	0.096	39.20
high	17	319.78	-11.914	-26.093	2.265	0.100	0.080	34.50
Total	27	321.31	-14.408	-24.764	-4.051	0.006	0.04	34.90
**Ile, µmol/L**								
breed								
Holstein	25	89.348	5.833	3.076	8.590	<0.001	0.704	0
others	5	101.1	5.335	-6.192	16.862	0.364	0.042	59.60
Total	30	91.31	5.641	3.017	8.264	<0.001	0.422	2.80
parity								
multiparous	17	86.65	3.605	0.082	7.127	0.045	0.766	0
mix	13	97.4	7.577	3.120	12.035	0.001	0.23	21.10
Total	30	91.31	5.641	3.017	8.264	<0.001	0.422	2.80
DIM								
pre	7	86.01	2.263	-4.503	9.028	0.512	0.216	27.80
early	16	88.86	6.696	3.147	10.246	<0.001	0.417	3.10
mid	7	102.2	5.567	0.208	10.926	0.042	0.563	0
Total	30	91.31	5.641	3.017	8.264	<0.001	0.422	2.80
basic feed								
corn silage	21	89.55	6.707	3.635	9.778	<0.001	0.7	0
grass silage								
mix	7	106.44	6.344	0.869	11.819	0.023	0.401	3.30
Total	28	93.77	6.634	3.971	9.298	<0.001	0.713	0
CP								
low	11	93.83	8.202	3.993	12.411	<0.001	0.904	0
high	19	89.85	3.664	-0.125	7.454	0.058	0.195	21.30
Total	30	91.31	5.641	3.017	8.264	<0.001	0.422	2.80
**Leu, µmol/L**								
breed								
Holstein	26	145.53	5.543	1.751	9.336	0.004	0.432	2.10
others	5	146.12	3.514	-12.741	19.769	0.672	0.054	56.90
Total	31	145.62	5.325	1.438	9.212	0.007	0.239	14.50
parity								
multiparous	18	156.94	5.290	0.244	10.336	0.04	0.369	7.20
mix	13	129.95	5.332	-1.026	11.690	0.100	0.158	28.50
Total	31	145.62	5.325	1.438	9.212	0.007	0.239	14.50
DIM								
pre	7	121.56	7.260	-2.129	16.649	0.130	0.09	45.20
early	17	158.49	6.193	0.551	11.835	0.031	0.358	8.20
mid	7	138.43	1.843	-4.804	8.490	0.587	0.512	0
Total	31	145.62	5.325	1.438	9.212	0.007	0.239	14.50
basic feed								

corn silage	22	150.5	4.860	0.543	9.176	0.027	0.517	0
grass silage								
mix	7	148.69	7.043	-1.066	15.152	0.089	0.299	17.10
Total	29	150.14	5.479	1.777	9.180	0.004	0.485	0
CP								
low	11	116.66	5.818	0.753	10.883	0.024	0.536	0
high	20	161.55	5.232	-0.806	11.270	0.089	0.129	27.00
Total	31	145.62	5.325	1.438	9.212	0.007	0.239	14.50
**Lys, µmol/L**								
breed								
Holstein	26	54.43	10.711	7.521	13.901	<0.001	0	60.70
others	5	79.48	5.821	-3.027	14.670	0.197	0.131	46.60
Total	31	58.47	10.112	7.119	13.106	<0.001	0	58.60
parity								
multiparous	18	54.7	12.880	8.460	17.299	<0.001	0	66.10
mix	13	63.69	6.517	3.418	9.615	<0.001	0.291	15.20
Total	31	58.47	10.112	7.119	13.106	<0.001	0	58.60
DIM								
pre	7	58.14	10.164	3.214	17.114	0.004	0.025	58.50
early	17	54.26	7.656	4.070	11.242	<0.001	0.003	55.80
mid	7	69	17.700	10.996	24.405	<0.001	0.078	47.10
Total	31	58.47	10.112	7.119	13.106	<0.001	0	58.60
basic feed								
corn silage	22	57.21	10.440	6.939	13.940	<0.001	0	57.80
grass silage								
mix	7	62.14	5.876	2.474	9.278	0.001	0.488	0
Total	29	58.4	9.414	6.604	12.225	<0.001	0	53.60
CP								
low	11	61.16	12.938	7.514	18.362	<0.001	0.007	58.90
high	20	56.985	8.722	5.047	12.397	<0.001	0	59.80
Total	31	58.47	10.112	7.119	13.106	<0.001	0	58.60
**Met, µmol/L**								
breed								
Holstein	26	17.49	8.475	6.150	10.800	<0.001	0	88.70
others	5	20.24	7.131	1.586	12.677	0.012	0	91.20
Total	31	17.93	8.242	6.139	10.345	<0.001	0	88.80
parity								
multiparous	18	17.49	8.995	5.911	12.080	<0.001	0	89.00
mix	13	18.54	7.308	4.313	10.303	<0.001	0	89.30
Total	31	17.93	8.242	6.139	10.345	<0.001	0	88.80
DIM								
pre	7	18.4	4.107	-0.480	8.694	0.079	0	86.50
early	17	17.99	8.757	6.118	11.397	<0.001	0	90.10
mid	7	17.33	11.179	5.976	16.382	<0.001	0	83.00

Total	31	17.93	8.242	6.139	10.345	<0.001	0	88.80
basic feed								
corn silage	22	17.5	9.577	6.915	12.240	<0.001	0	89.30
grass silage								
mix	7	20.47	6.671	2.972	10.370	<0.001	0	88.50
Total	29	18.22	8.805	6.679	10.931	<0.001	0	88.80
CP								
low	11	16.92	8.347	5.346	11.349	<0.001	0	83.00
high	20	18.49	8.147	5.265	11.028	<0.001	0	90.90
Total	31	17.93	8.242	6.139	10.345	<0.001	0	88.80
**Val, µmol/L**								
breed								
Holstein	26	204.61	7.799	-1.269	16.867	0.092	0	61.40
others	5	228.38	2.348	-19.774	24.471	0.835	0.113	46.50
Total	31	208.44	7.072	-1.244	15.387	0.096	0	58.80
parity								
multiparous	18	197.82	6.213	-4.826	17.253	0.270	0	63.70
mix	13	223.15	8.323	-4.805	21.452	0.214	0.011	53.90
Total	31	208.44	7.072	-1.244	15.387	0.096	0	58.80
DIM								
pre	7	223.04	10.49	0.958	20.022	0.031	0.545	0.00
early	17	206.9	9.630	-0.353	19.613	0.059	0.043	40.40
mid	7	197.59	-0.213	-27.973	27.546	0.988	0	84.20
Total	31	208.44	7.072	-1.244	15.387	0.096	0	58.80
basic feed								
corn silage	22	202.58	6.712	-4.675	18.098	0.248	0	68.20
grass silage								
mix	7	235.1	3.610	-8.535	15.756	0.560	0.73	0
Total	29	210.43	6.277	-2.731	15.284	0.172	0	60.00
CP								
low	11	199.2	3.928	-13.948	21.805	0.667	0	75.40
high	20	213.525	9.396	0.890	17.903	0.030	0.043	38.30
Total	31	208.44	7.072	-1.244	15.387	0.096	0	58.80

Table S4 Parameters of the models between supplementary RPML and performance of dairy cows

	Intercept		A (d-Met)		B (d-Lys)		AB (d-Met×d-Lys)		A^2		B^2		Models	
	Coefficient	P-value	Coefficient	P-value	Coefficient	P-value	Coefficient	P-value	Coefficient	P-value	Coefficient	P-value	P-value	$AdjR^2$
Milk Yield, kg/d	0.4566	0.1253	-0.0633	0.1037	0.0156	0.1906	0.0004	0.3401	0.0013	0.1595	-0.0002	0.03189	0.07893	0.0922
Milk Protein, kg/d	0.4338	0.09242	-0.051	0.07793	0.0195	0.02208	0.0005	0.08434	0.0011	0.1107	-0.0003	0.000399	0.005411	0.1429
Milk Protein, %	0.6917	0.02176	-0.0244	0.4648	-0.0116	0.3211	0.0007	0.2859	0.0003	0.7722	0	0.826	0.4927	-0.1203
Milk Fat, kg/d	0.3463	0.1274	-0.0504	0.04182	0.0114	0.1113	0.0003	0.1336	0.001	0.08515	-0.0001	0.02123	0.03506	0.0506
Milk Fat, %	0.2462	0.1885	-0.0446	0.02756	0.0101	0.09904	0.0002	0.3394	0.0008	0.09233	-0.0001	0.1521	0.01526	0.4966
3.5%FCM, kg/d	0.6428	0.02498	-0.0861	0.002085	0.0176	0.08584	0.0003	0.2476	0.0018	0.007085	-0.0002	0.01898	0.008812	0.2508
Milk Lactose, kg/d	0.6032	0.03099	-0.0851	0.01947	0.0018	0.8439	-0.0006	0.1825	0.0036	0.0002053	0	0.6412	0.0003772	0.6262
DMI, kg/d	0.3874	0.1432	-0.0859	0.003685	0.0163	0.08957	0.0003	0.2361	0.0021	0.005687	-0.0002	0.02531	0.001398	-0.1727
Ile, µmol/L	0.9419	0.0005581	-0.0573	0.05194	-0.0133	0.2056	0.0001	0.7725	0.0011	0.09906	0.0001	0.3788	0.1375	0.4347
Lys, µmol/L	0.7539	0.03608	-0.066	0.1032	0.0171	0.215	-0.0005	0.3439	0.002	0.03218	0	0.7788	0.2702	-0.0376
Met, µmol/L	0.308	0.6122	0.1885	0.003635	-0.0455	0.04576	0.0017	0.09696	-0.0045	0.00422	-0.0001	0.814	0.01328	-0.1868

Note:Milk yield (kg/d) used data from cows of Holstein, multiparous, early-lactation stage, fed corn silage and high CP diet;

Milk Protein (kg/d) used data from cows of Holstein, multiparous, early-lactation stage;

Milk protein (%) used data from cows of Holstein,multiparous, early-lactation stage, fed corn silage;

Milk fat (kg/d) used data from cows of Holstein, multiparous, prepartum and early-lactation stage, fed high-protein grass silage;

Milk fat (%) used data from cows of Holstein, fed high-protein mix silage;

3.5% FCM (kg/d) used data from cows of Holstein, multiparous, prepartum, fed high-protein grass silage;

Milk lactose (kg/d) used data from cows of Holstein;

DMI (kg/d) used data from cows of Holstein, prepartum;

Ile (µmol/L) used data from cows of Holstein, early- and mid-lactation stage, fed low-protein diet;

Lys (µmol/L) used data from cows of Holstein;

Met (µmol/L) used data from cows of Holstein, early- and mid-lactation stage;
