# Supplementary material for: Effect of Dietary Rumen-Protected Methionine and Lysine on Performance and Plasma Amino Acid of Dairy Cows: A Meta-Analysis
Source: Animals (Basel). 2026 Oct 31;16(21):3442. doi: 10.3390/ani16213442 (PMC13761621; doi:10.3390/ani16213442)
Supplement: Supplementary file 1 [file animals-16-03442-s001.zip › 3 Figure S1,S2-20260919-1019-1028.pdf]

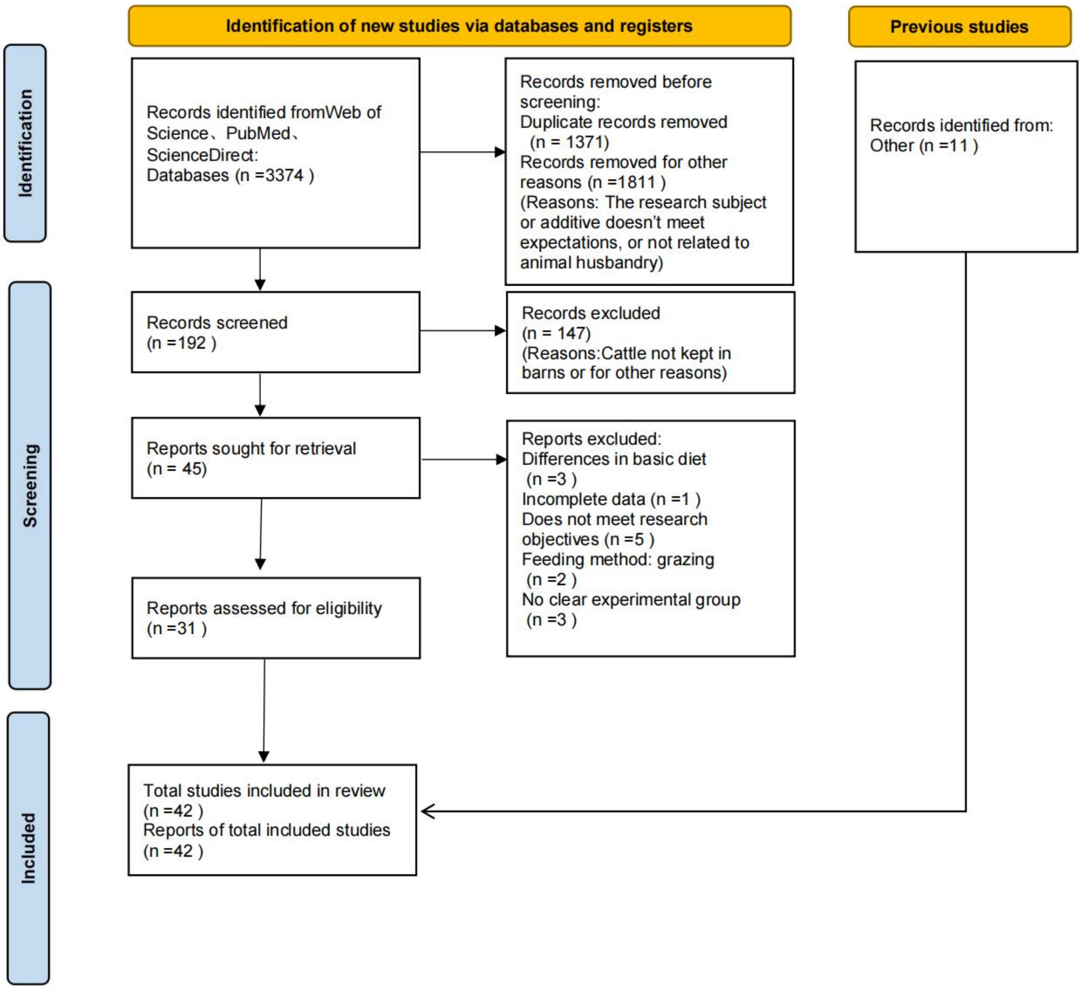

Figure S1. The flowchart of the search and selection strategies of eligible studies.

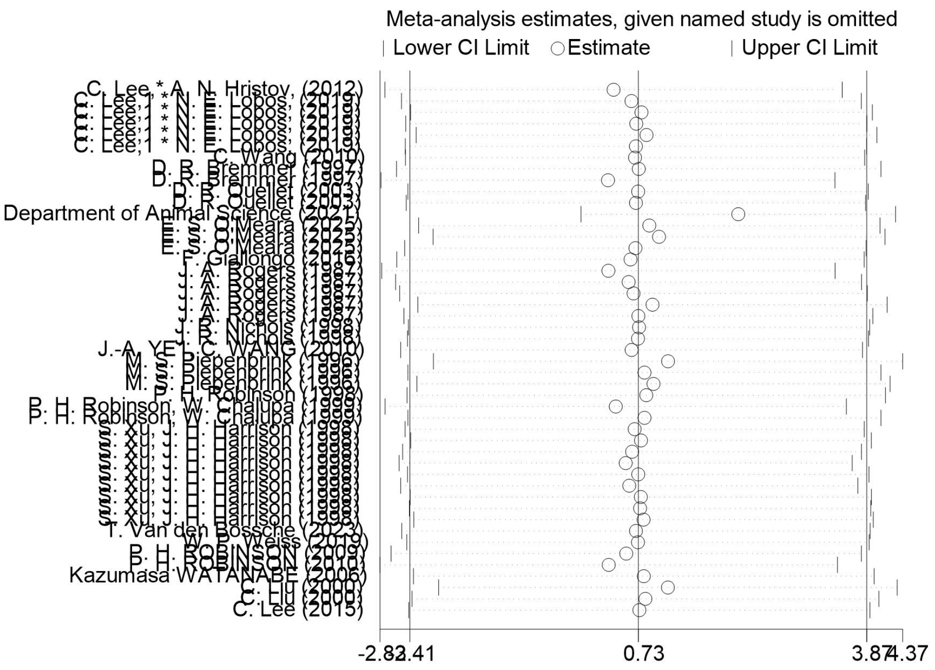

(1) BW,kg

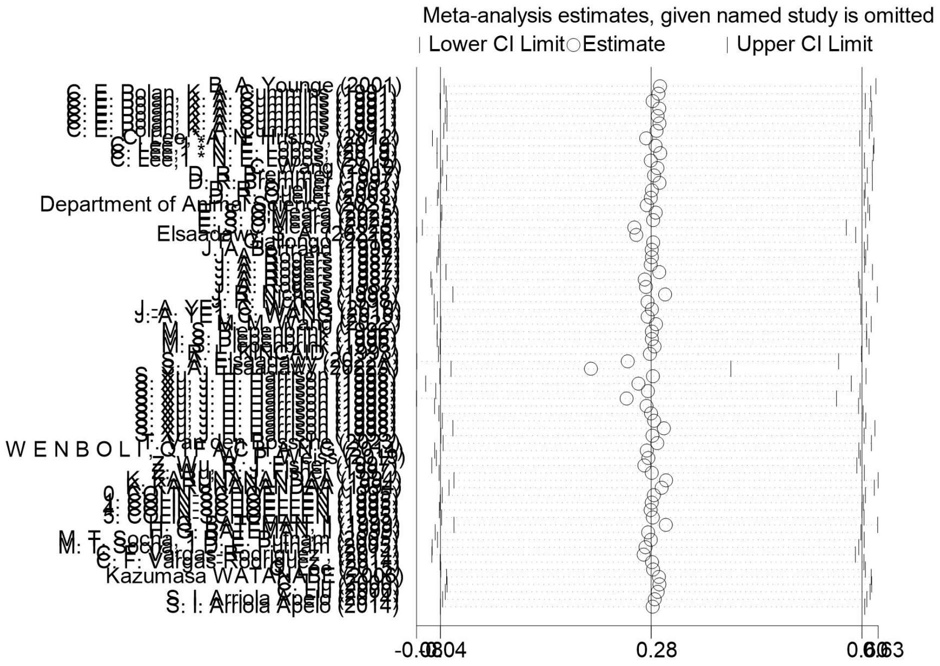

(2) DMI,kg/d

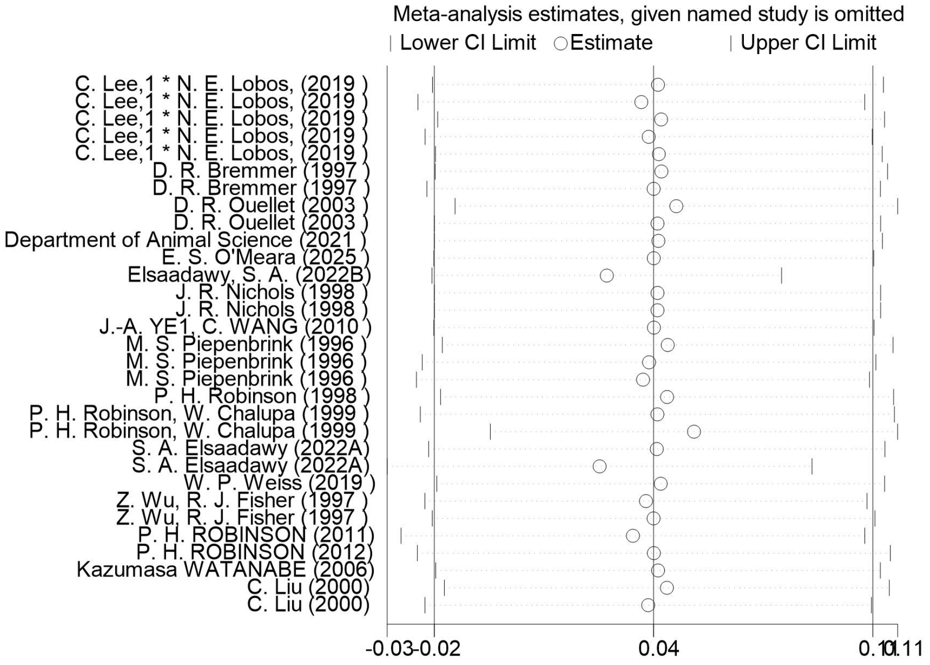

(3) BCS

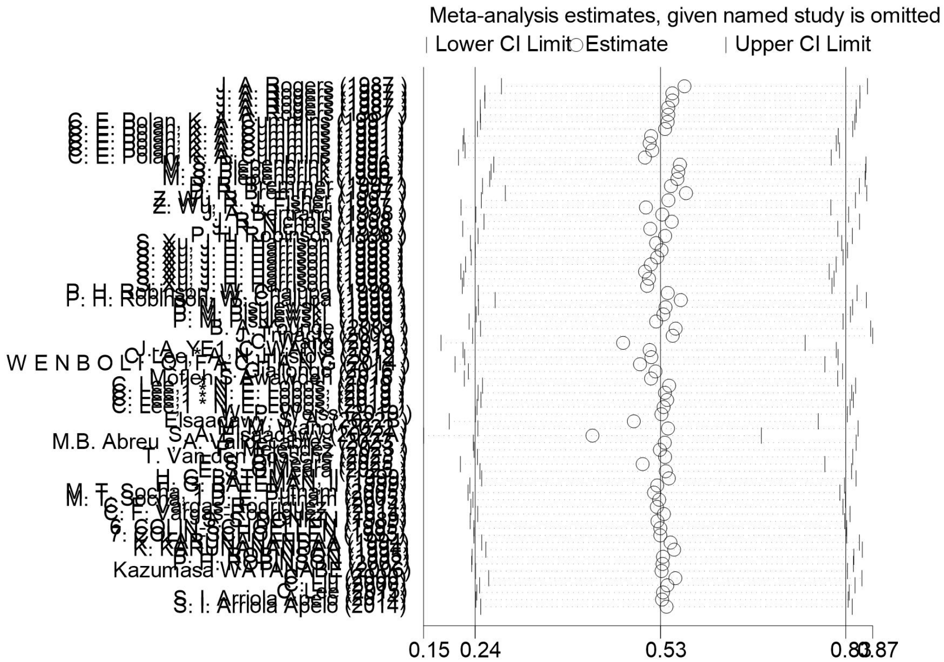

(4) Milk Yield,kg/d

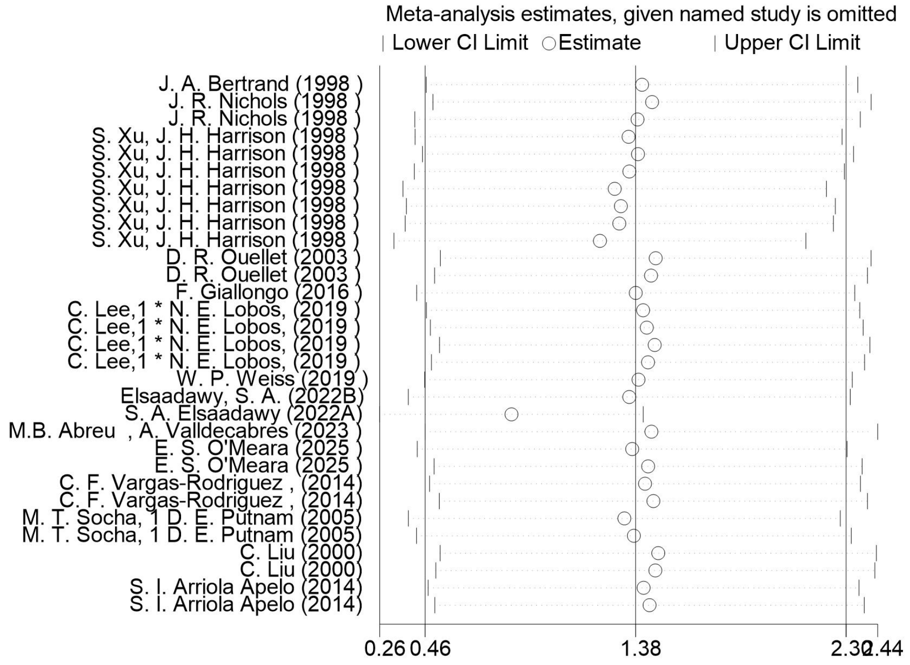

(5) ECM,kg/d

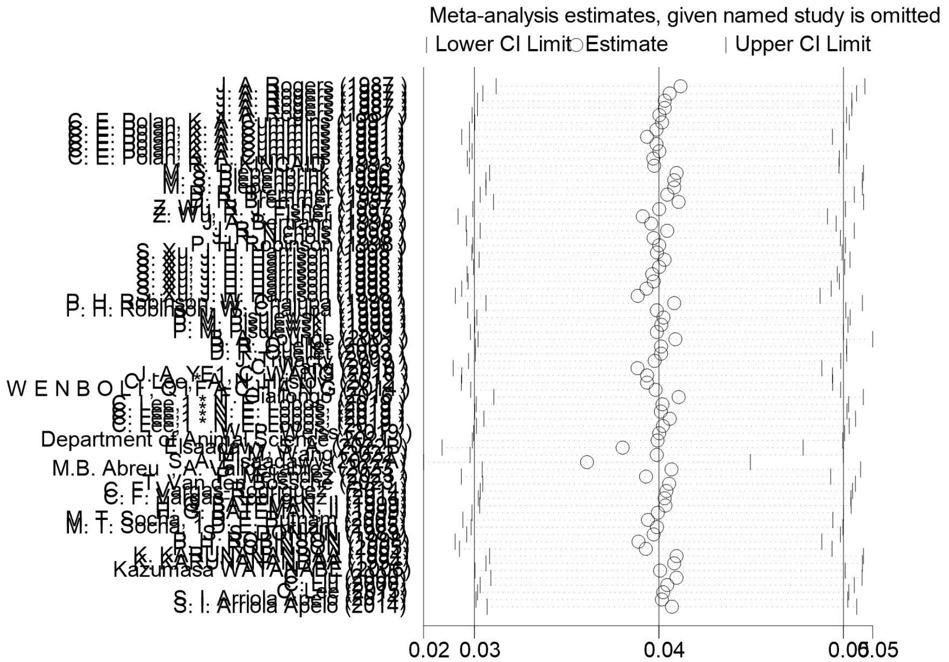

(6) Milk Protein,kg/d

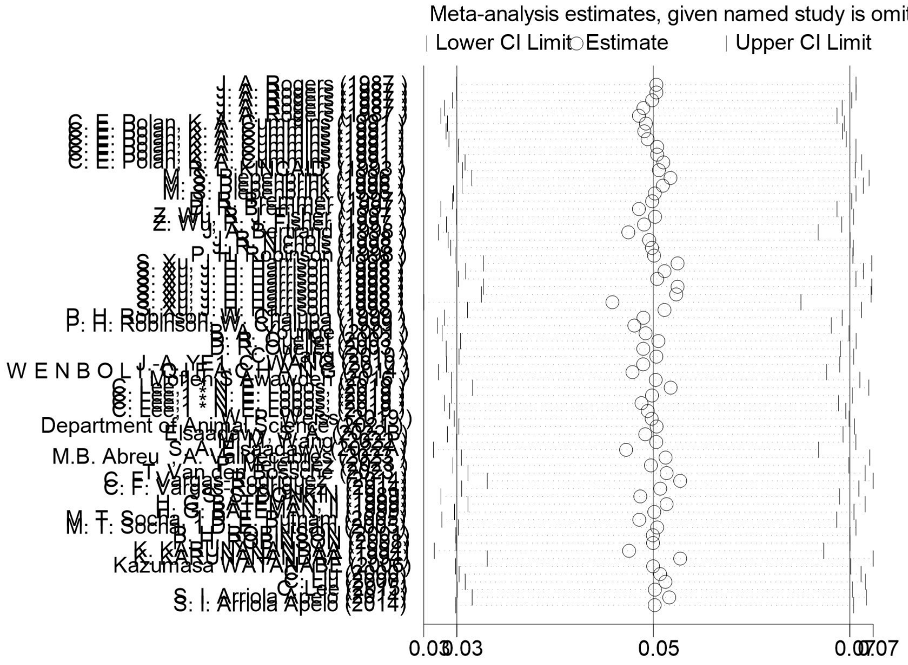

(7) Milk Protein,%

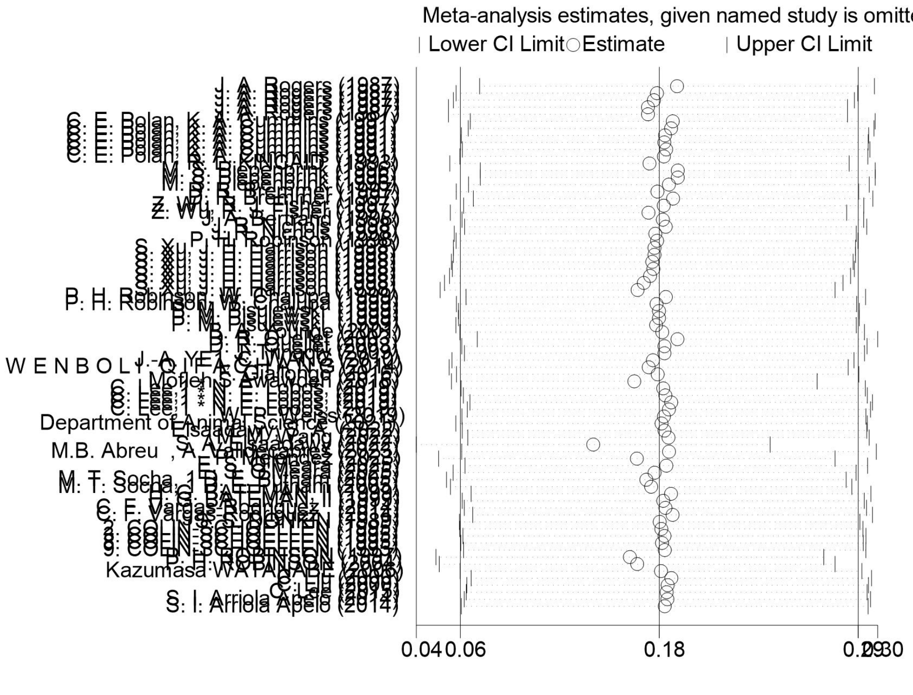

(8) Milk Fat,kg/d

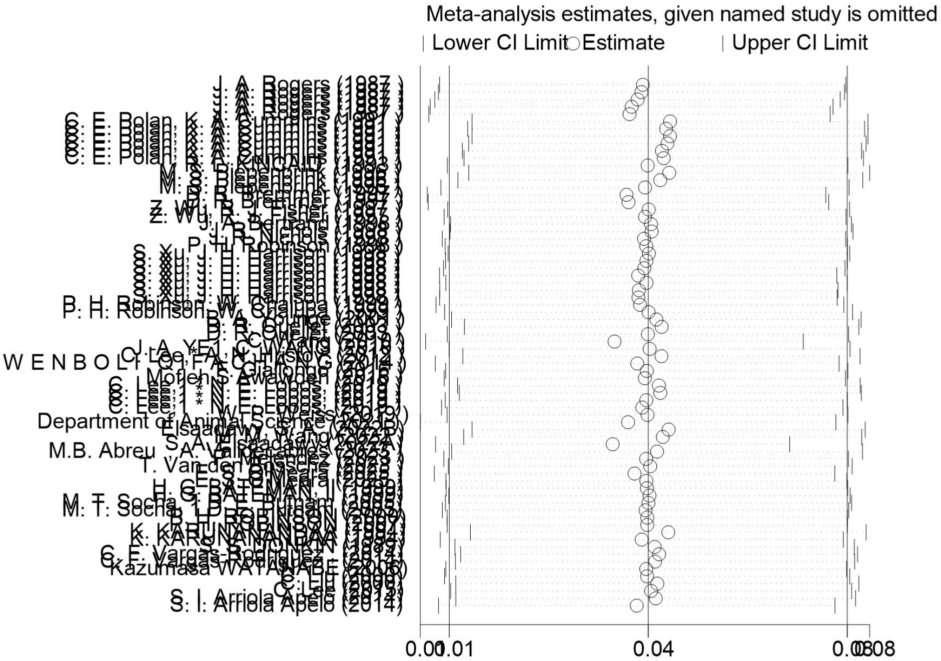

(9) Milk Fat,%

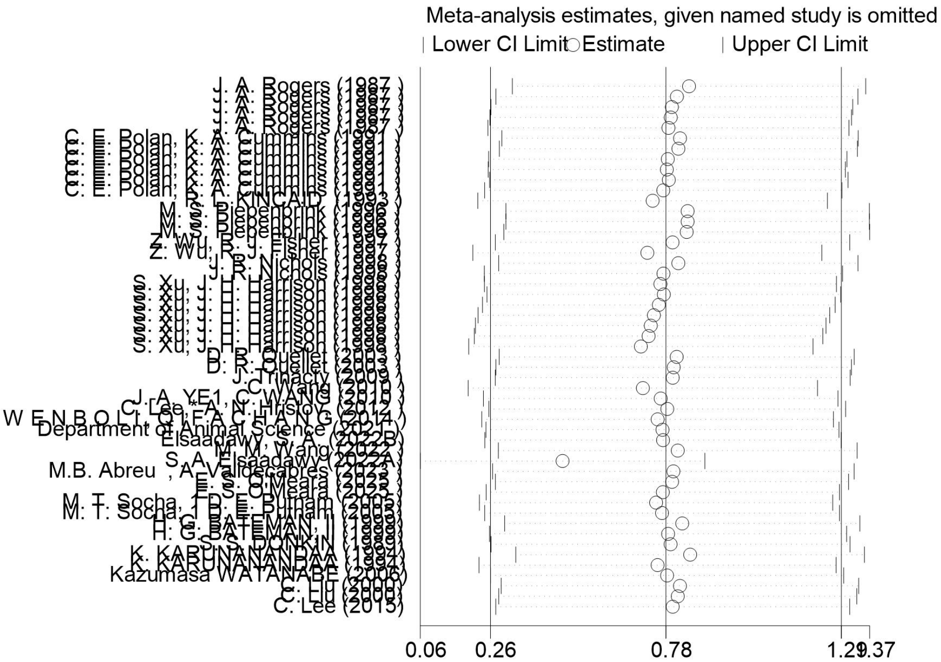

(10) 3.5%FCM,kg/d

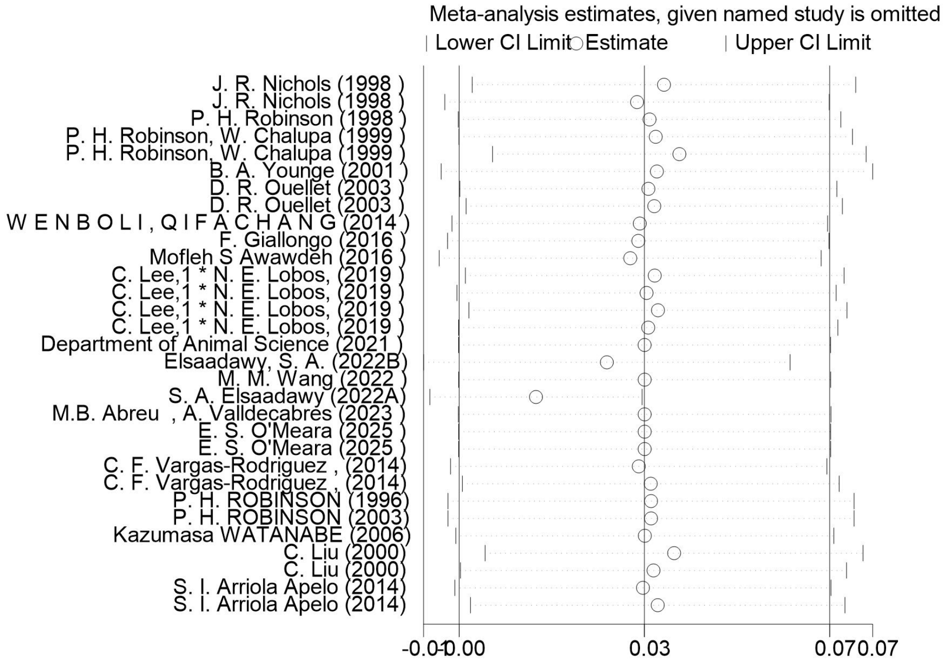

(11) Milk Lactose,kg/d

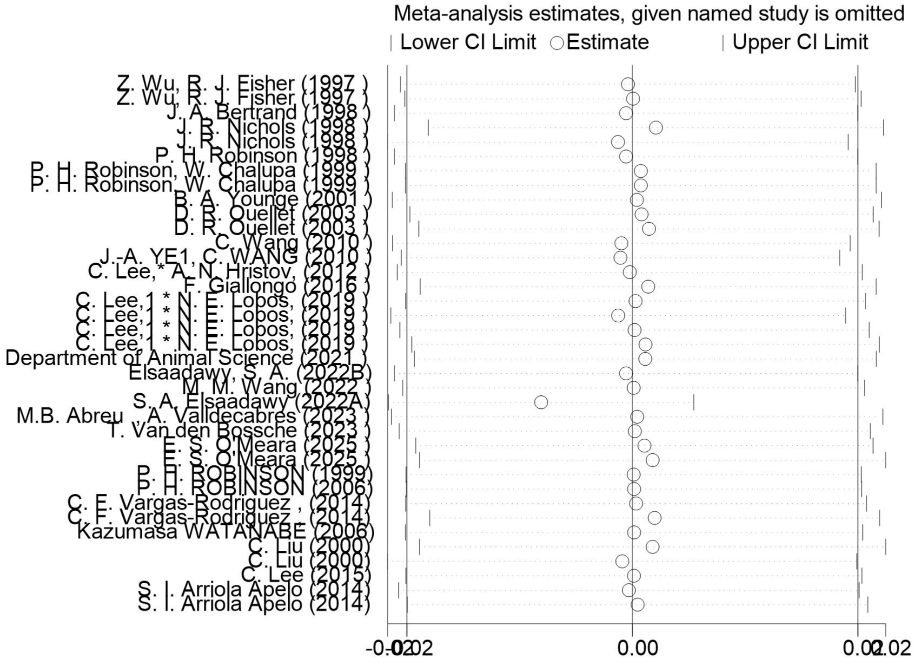

(12) Milk Lactose,%

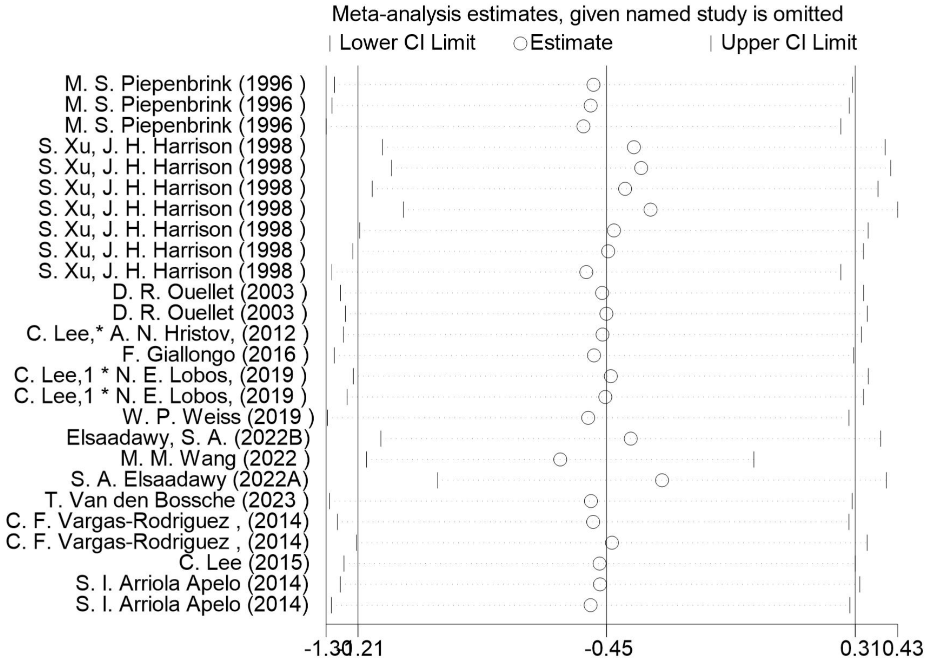

(13) MUN,mg/dL

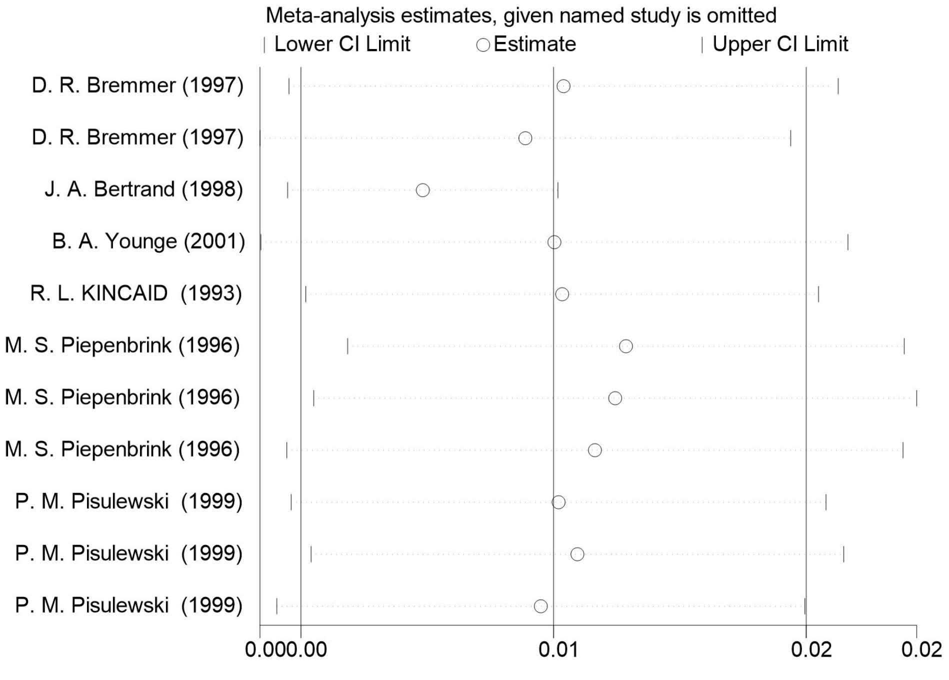

(14) Total N,%

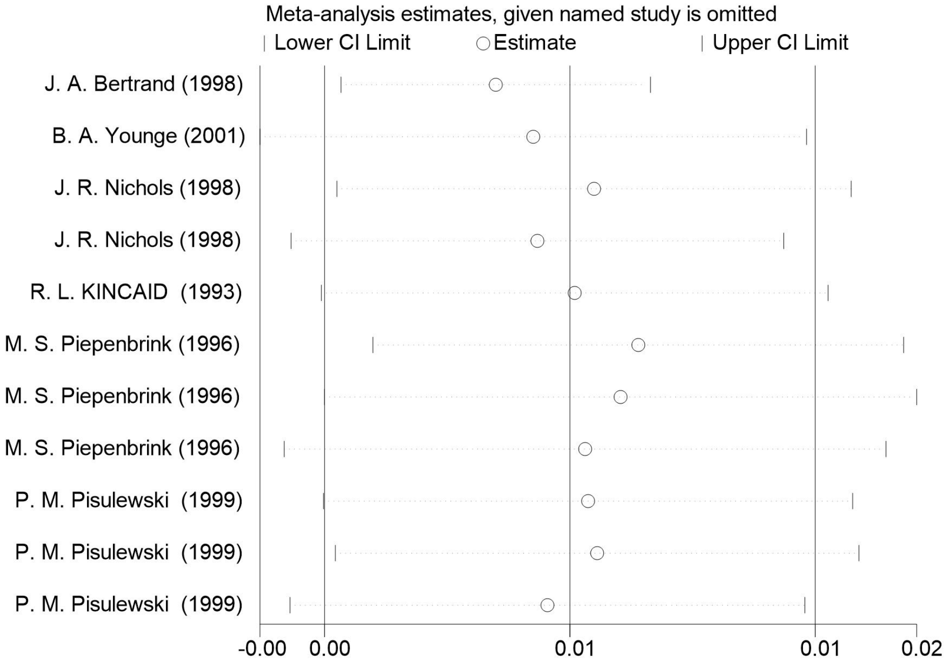

(15) Casein N,%

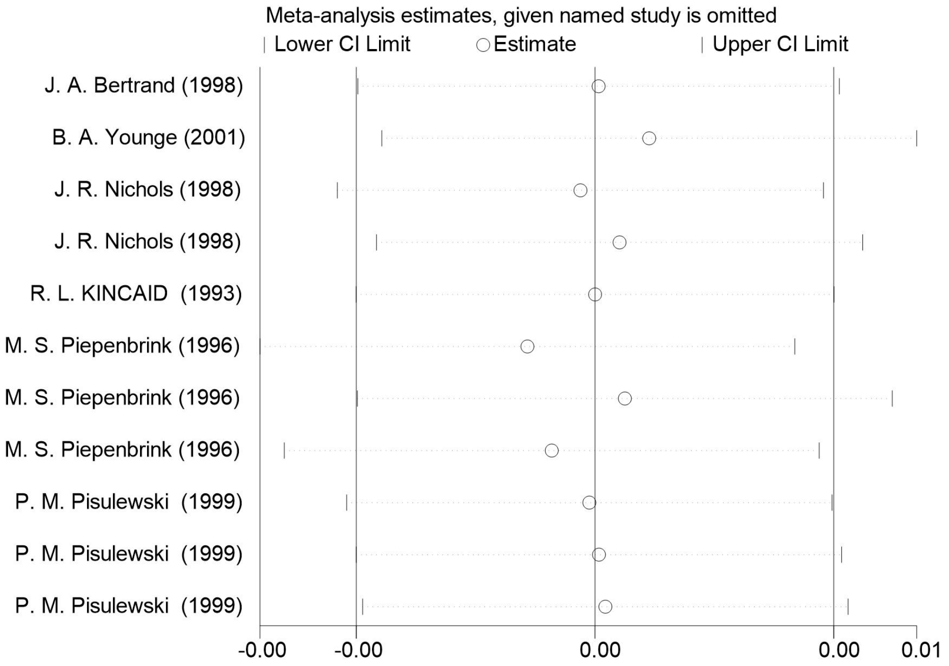

(16) Whey N,%

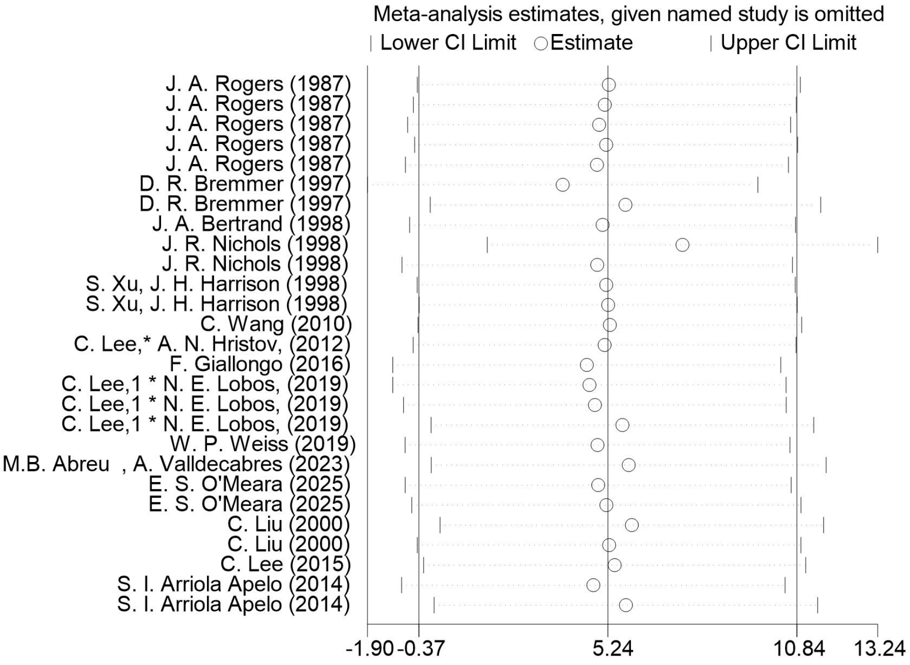

(17) Ala,μmol/L

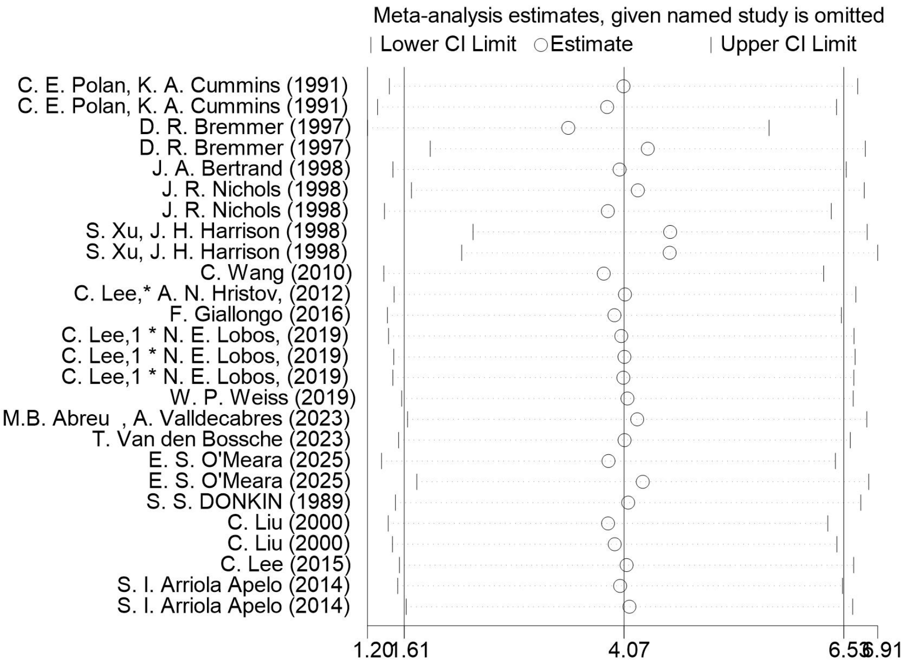

(18) Arg,μmol/L

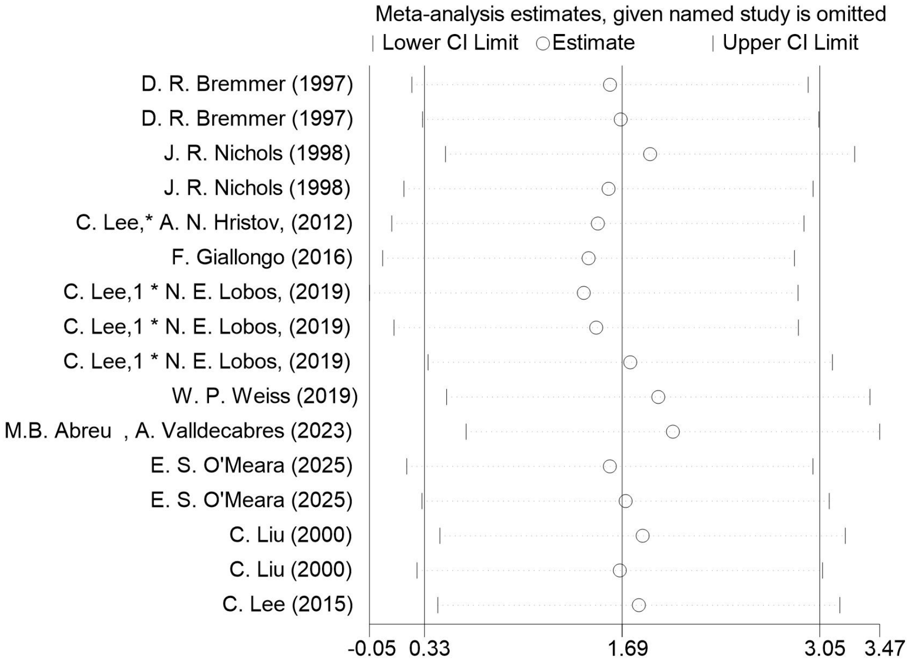

(19) Asn,μmol/L

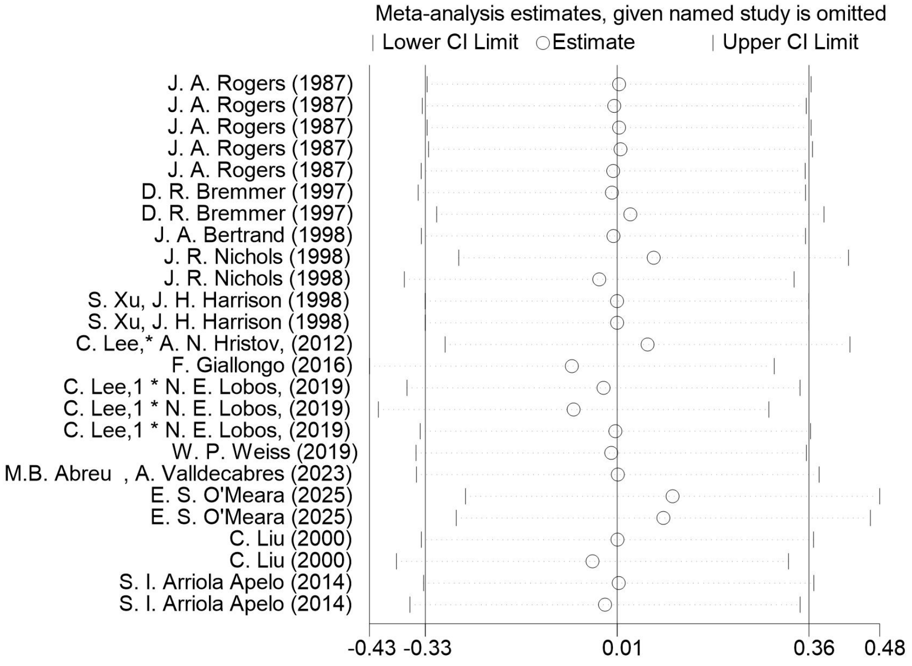

(20) Asp,μmol/L

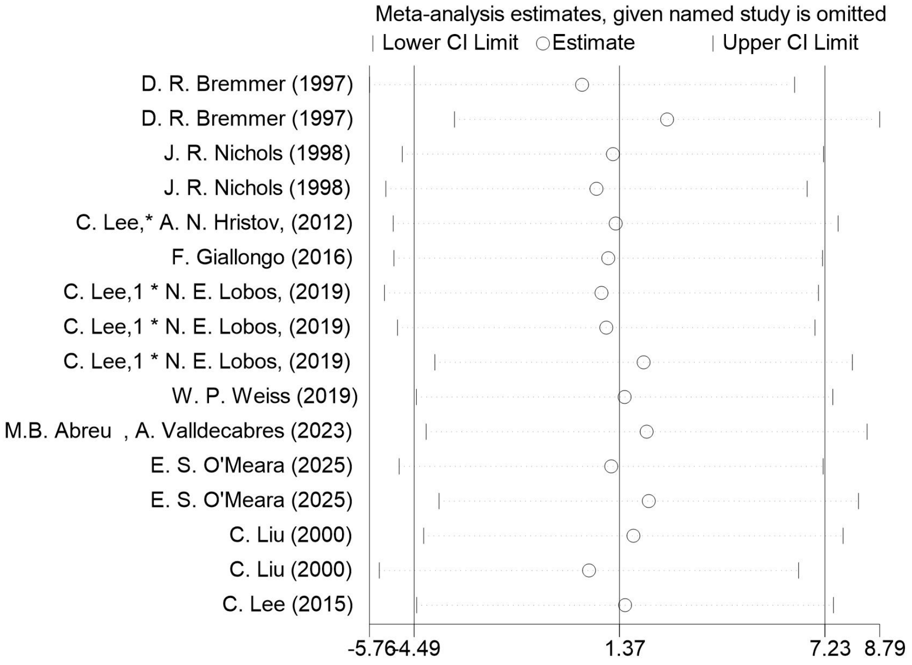

(21) Gln,μmol/L

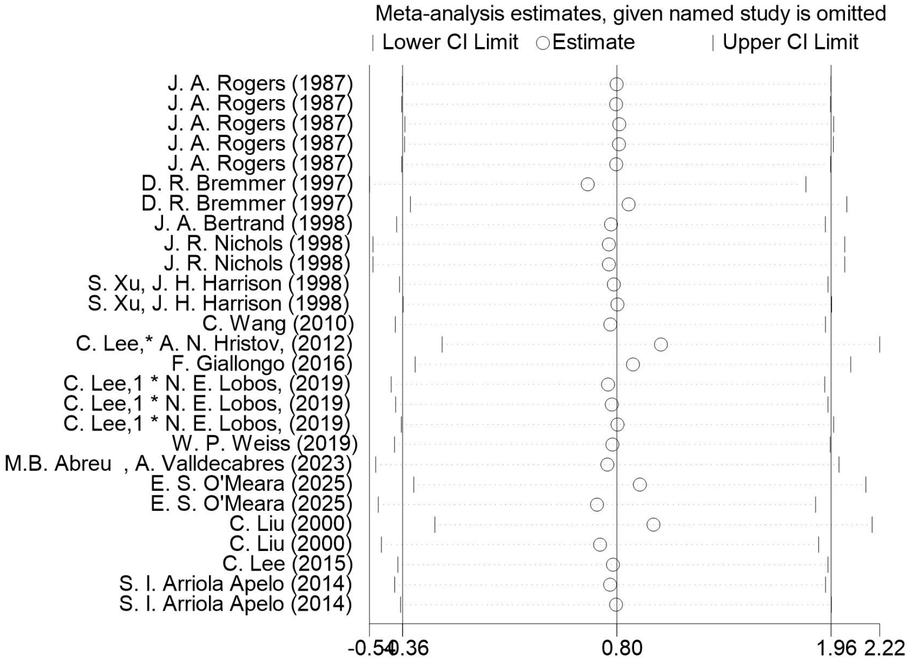

(22) Glu,μmol/L

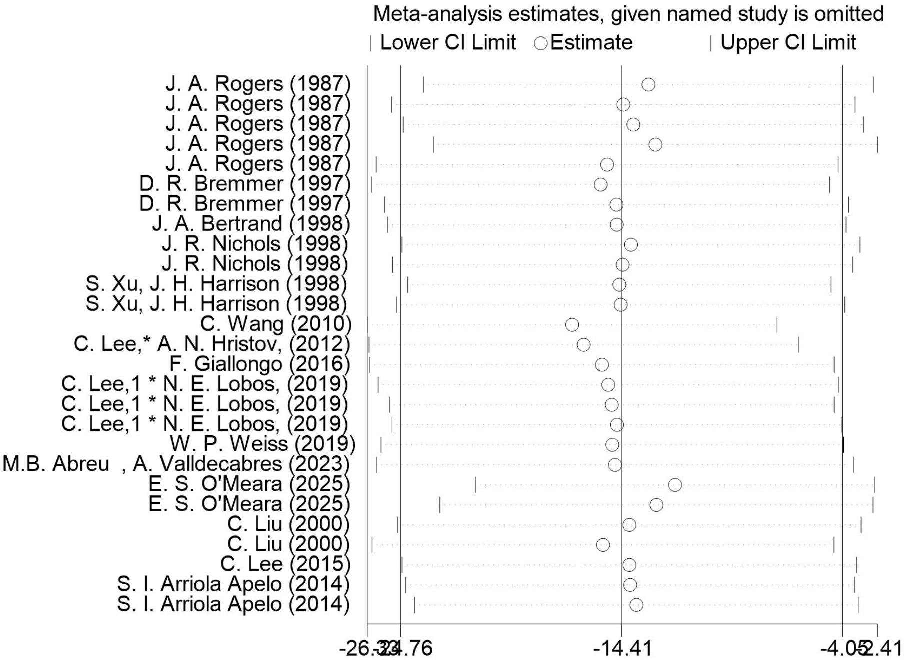

(23) Gly,μmol/L

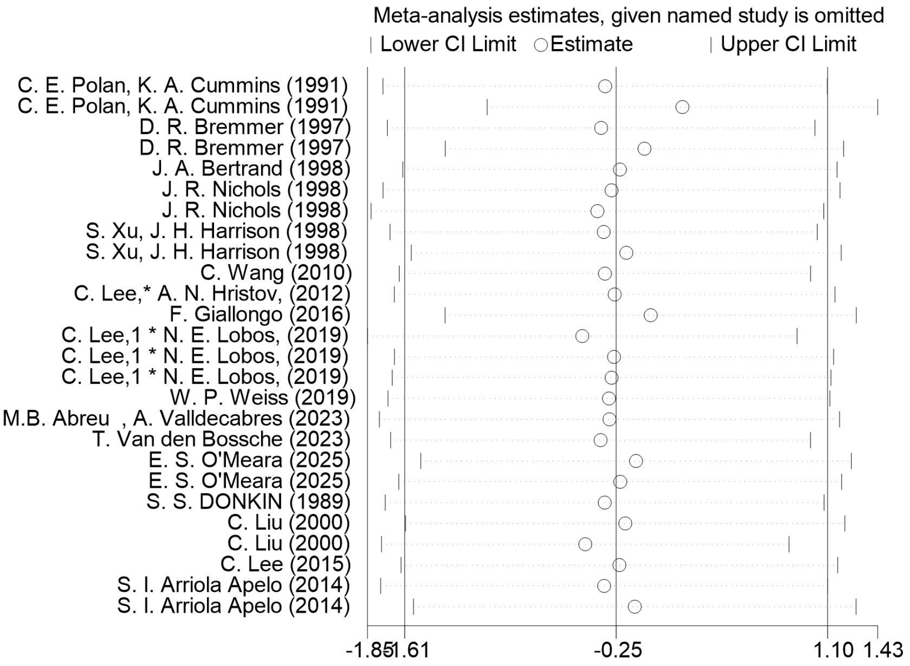

(24) His,μmol/L

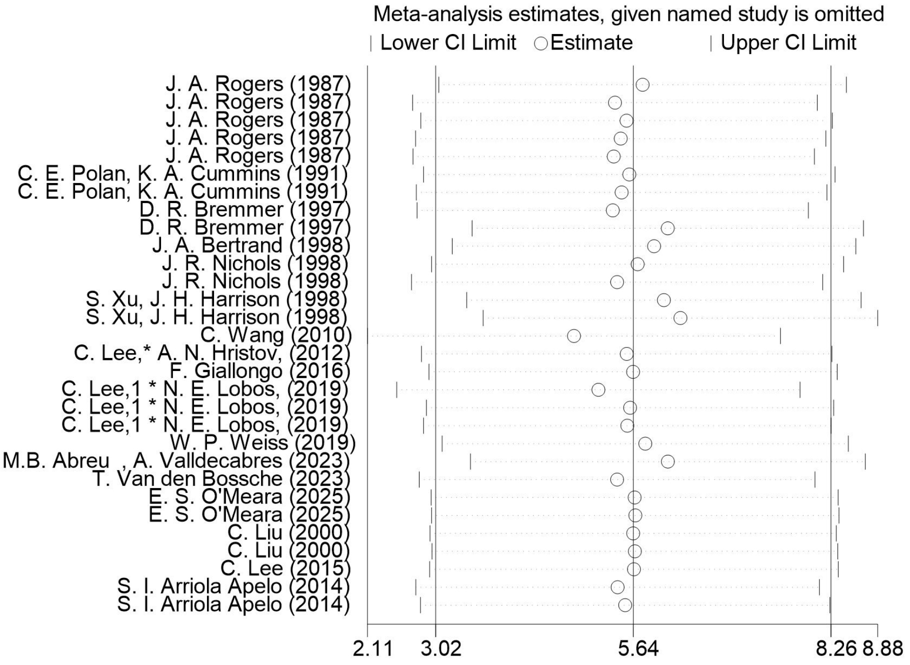

(25) Ile,μmol/L

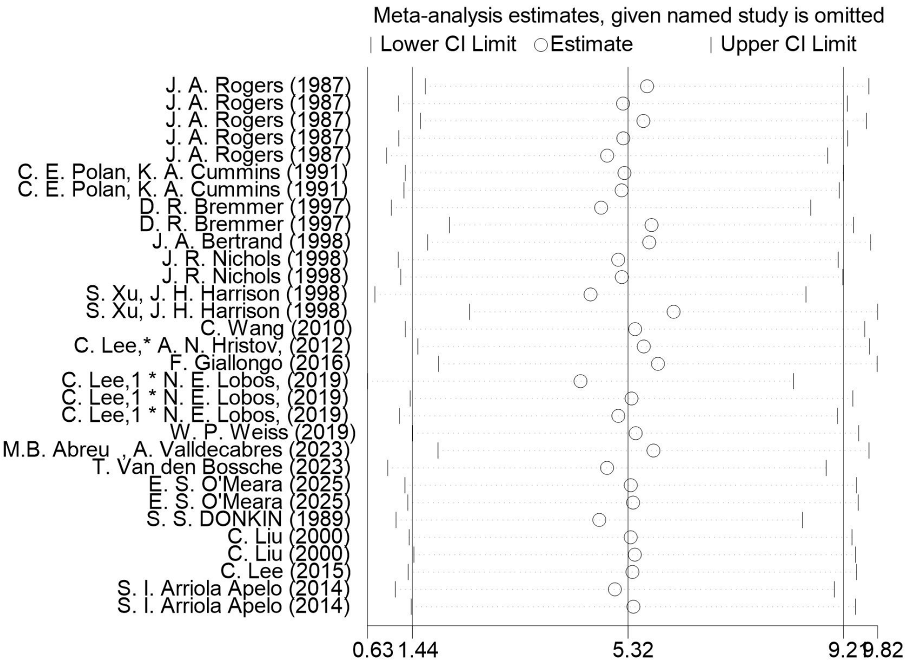

(26) Leu,μmol/L

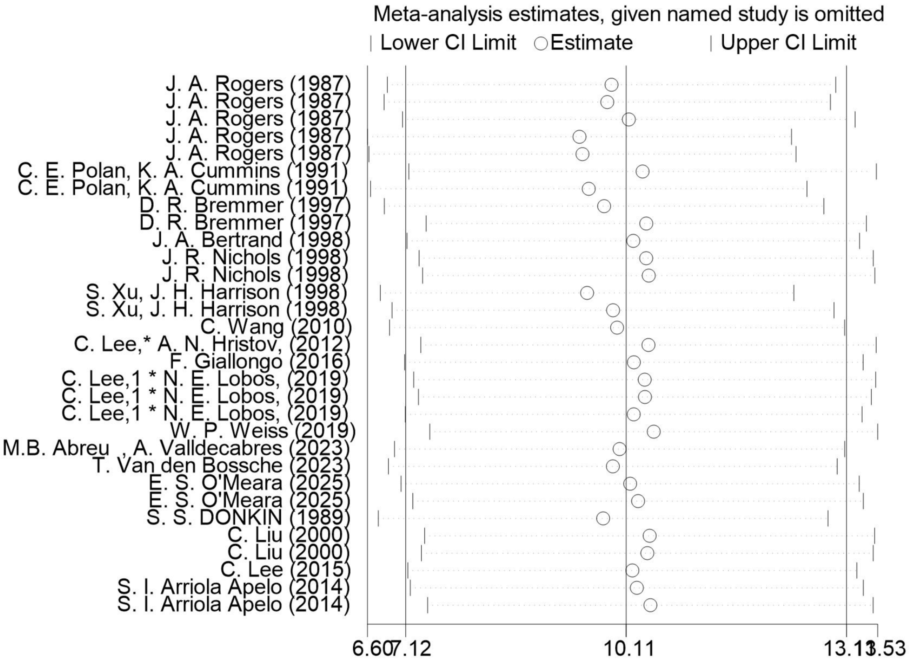

(27) Lys,μmol/L

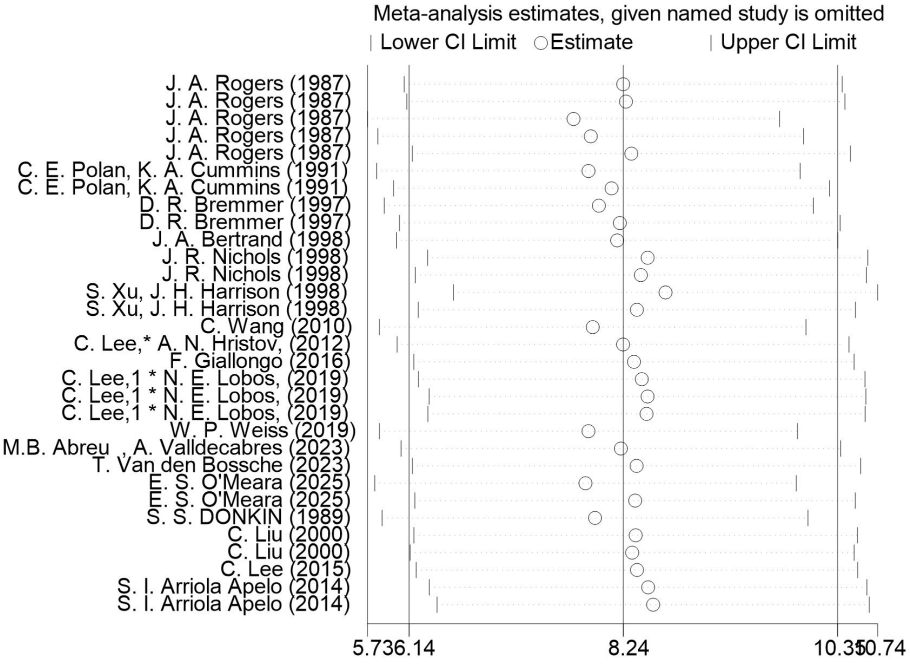

(28) Met,μmol/L

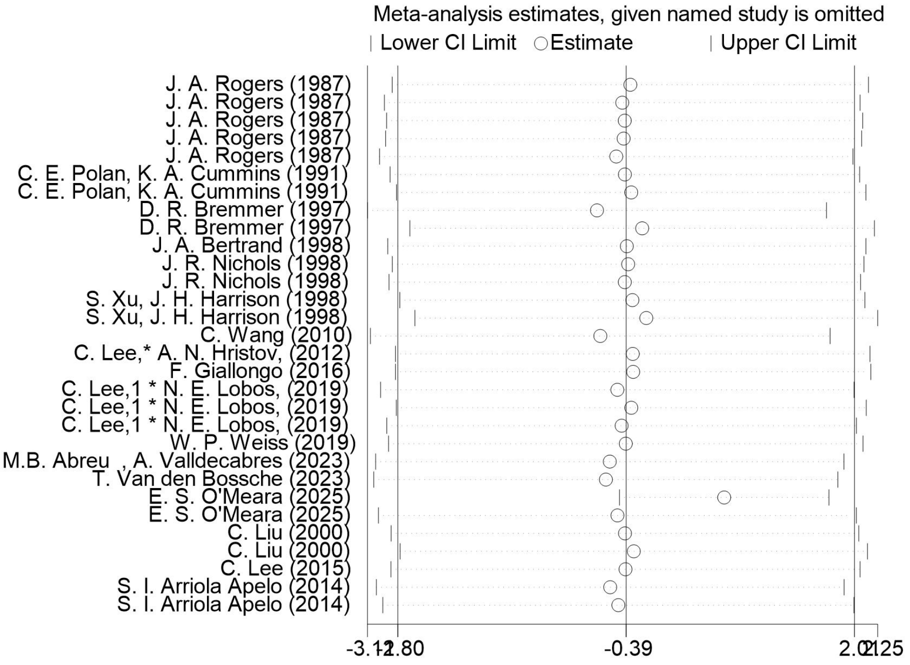

(29) Phe,μmol/L

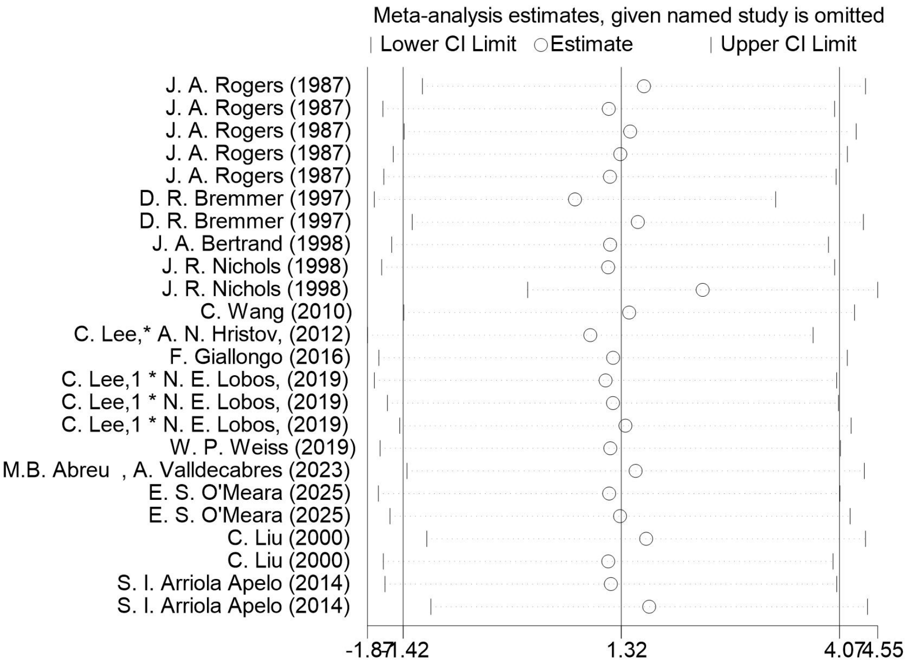

(30) Pro,μmol/L

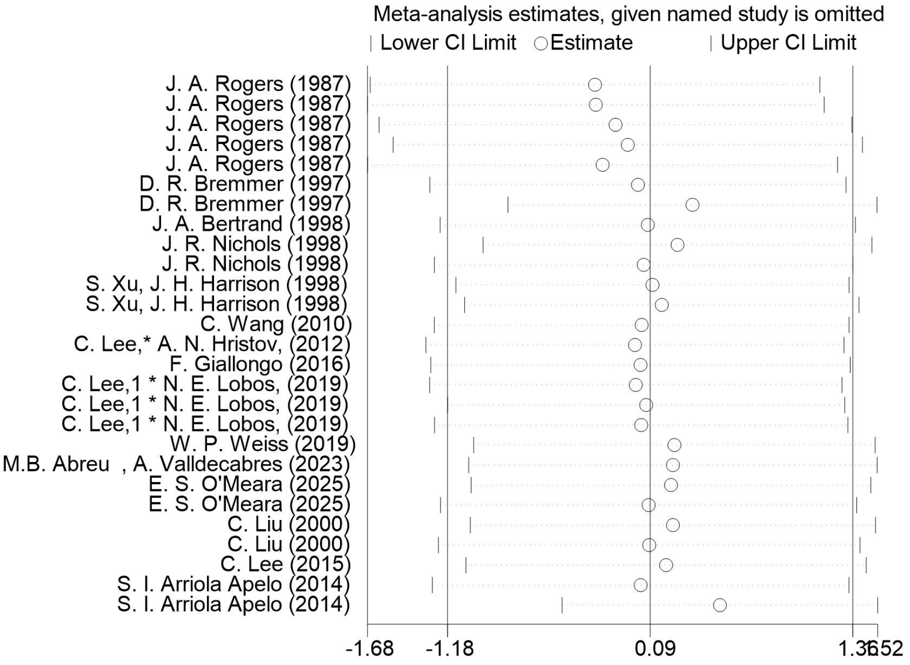

(31) Ser,μmol/L

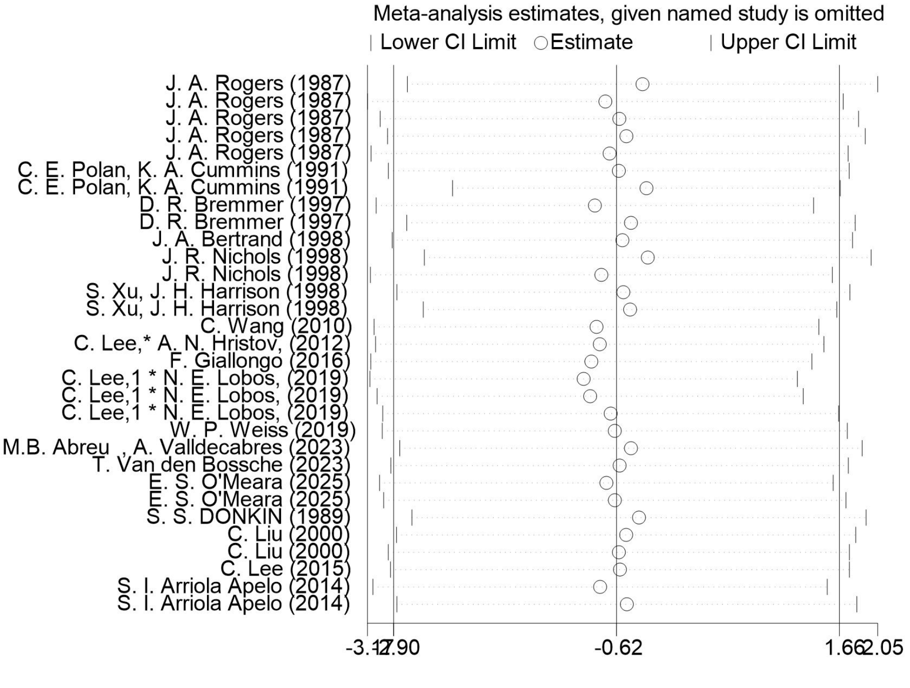

(32) Thr,μmol/L

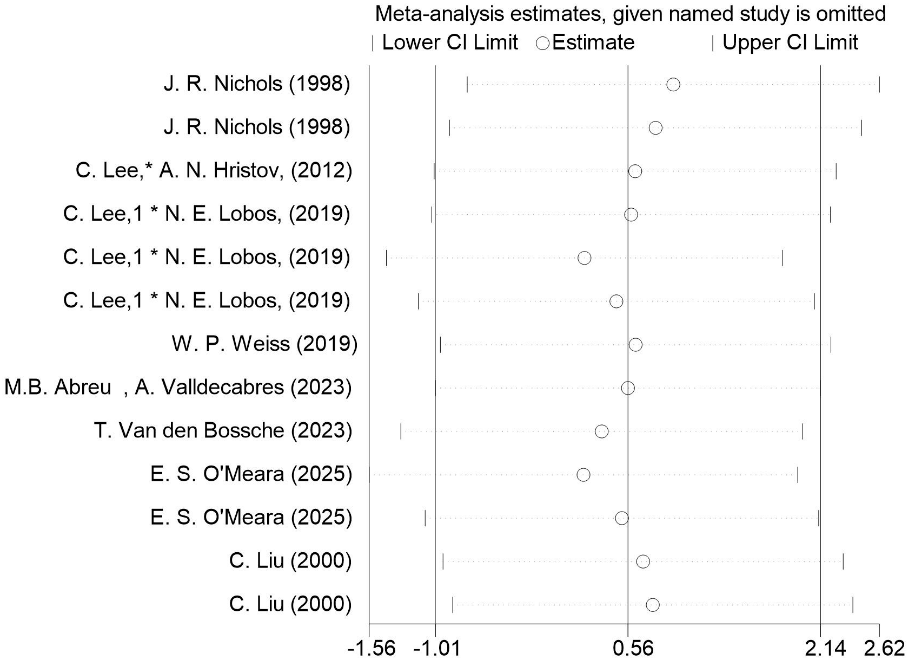

(33) Trp,μmol/L

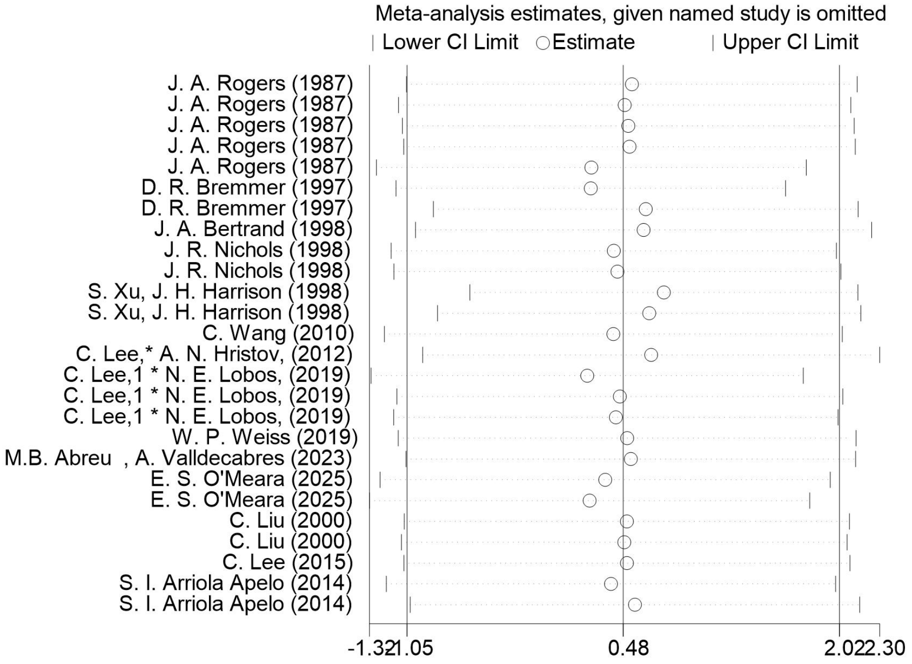

(34) Tyr,μmol/L

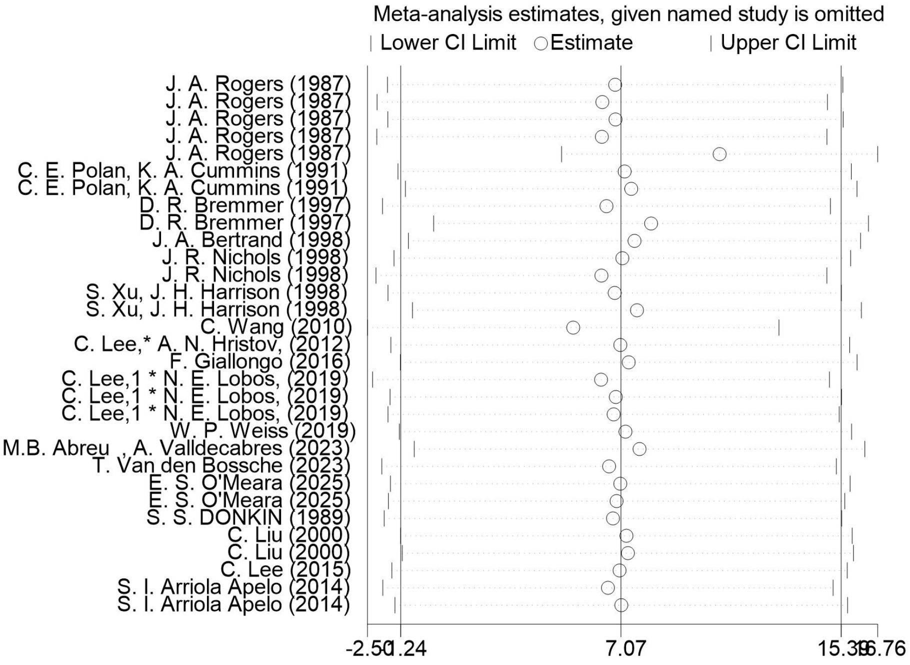

(35) Val,μmol/L

Figure S2. Sensitive analysis results.(1) BW (body weight), kg; (2) DMI (dry matter intake), kg/d; (3) BCS (body condition score); (4) Milk Yield, kg/d; (5) ECM (energy-corrected milk), kg/d; (6) Milk Protein, kg/d; (7) Milk Protein, %; (8) Milk Fat, kg/d; (9) Milk Fat, %; (10) 3.5%FCM (fat corrected milk), kg/d; (11) Milk Lactose, kg/d; (12) Milk Lactose, %; (13) MUN (milk urea nitrogen), mg/dL; (14) Total N (nitrogen), %; (15) Casein N, %; (16) Whey N, %; (17) Ala, μmol/L; (18) Arg, μmol/L; (19) Asn, μmol/L; (20) Asp, μmol/L; (21) Gln, μmol/L; (22) Glu, μmol/L; (23) Gly, μmol/L; (24) His, μmol/L; (25) Ile, μmol/L; (26) Leu, μmol/L; (27) Lys, μmol/L; (28) Met, μmol/L; (29) Phe, μmol/L; (30) Pro, μmol/L; (31) Ser, μmol/L; (32) Thr, μmol/L; (33) Trp, μmol/L; (34) Tyr, μmol/L; (35) Val, μmol/L.
